# 3-Dimensional ventricular electrical activation pattern assessed from a novel high-frequency electrocardiographic imaging technique: principles and clinical importance

**DOI:** 10.1038/s41598-021-90963-4

**Published:** 2021-06-01

**Authors:** Pavel Jurak, Laura R. Bear, Uyên Châu Nguyên, Ivo Viscor, Petr Andrla, Filip Plesinger, Josef Halamek, Vlastimil Vondra, Emma Abell, Matthijs J. M. Cluitmans, Rémi Dubois, Karol Curila, Pavel Leinveber, Frits W. Prinzen

**Affiliations:** 1 Institute of Scientific Instruments, The Czech Academy of Sciences, Kralovopolska 147, Brno, 635 00 Czech Republic; 2 IHU Liryc, Fondation Bordeaux Université, Pessac-Bordeaux, France; 3 Univ. Bordeaux, CRCTB, U1045, Bordeaux, France; 4 INSERM, CRCTB, U1045, Bordeaux, France; 5 Department of Physiology, Cardiovascular Research Institute Maastricht, Maastricht University Medical Center, Maastricht, The Netherlands; 6 Department of Cardiology, Cardiovascular Research Institute Maastricht, Maastricht University Medical Center, Maastricht, The Netherlands; 7 Cardiocenter, Department of Cardiology, 3rd Faculty of Medicine, Charles University and University Hospital Kralovske Vinohrady, Prague, Czech Republic; 8 International Clinical Research Center, St. Anne’s University Hospital, Brno, Czech Republic

**Keywords:** Cardiology, Cardiac device therapy

## Abstract

The study introduces and validates a novel high-frequency (100–400 Hz bandwidth, 2 kHz sampling frequency) electrocardiographic imaging (HFECGI) technique that measures intramural ventricular electrical activation. Ex-vivo experiments and clinical measurements were employed. Ex-vivo, two pig hearts were suspended in a human-torso shaped tank using surface tank electrodes, epicardial electrode sock, and plunge electrodes. We compared conventional epicardial electrocardiographic imaging (ECGI) with intramural activation by HFECGI and verified with sock and plunge electrodes. Clinical importance of HFECGI measurements was performed on 14 patients with variable conduction abnormalities. From 3 × 4 needle and 108 sock electrodes, 256 torso or 184 body surface electrodes records, transmural activation times, sock epicardial activation times, ECGI-derived activation times, and high-frequency activation times were computed. The ex-vivo transmural measurements showed that HFECGI measures intramural electrical activation, and ECGI-HFECGI activation times differences indicate endo-to-epi or epi-to-endo conduction direction. HFECGI-derived volumetric dyssynchrony was significantly lower than epicardial ECGI dyssynchrony. HFECGI dyssynchrony was able to distinguish between intraventricular conduction disturbance and bundle branch block patients.

## Introduction

Electrocardiographic imaging (ECGI) is a noninvasive cardiac electrical procedure that determines heart activity noninvasively from body-surface potential recordings through inverse reconstruction^1^. The most commonly used implementation of ECGI estimates epicardial potentials and epicardial electrical activation times (EAT)^2^. The accuracy of reconstruction of epicardial potentials has been invasively validated on animals and humans^3–5^.

ECGI has been used for multiple analyses of ventricular activation and repolarization during normal synchronous activation, pacing, arrhythmia^3,6^, and for purposes of ventricular dyssynchrony assessment^7^. With respect to the latter, ECGI was shown to improve the prediction of response to cardiac resynchronization therapy (CRT)^7–10^. Still, there are uncertainties in how to best assess the electrical substrate for cardiac pacing in clinical practice. The limitation of ECGI is epicardial activation only and frequent artificial clustering of activation times.

Recently, we have reported about high-frequency (HF, 150–400 Hz) and ultra-high-frequency (UHF, 150–1000 Hz) 12–14-lead ECG analysis to compute ventricular electrical dyssynchrony before and during pacing device implantation^11,12^. Using the HF ECG technique, we showed that the ventricular electrical delay computed from V1–V6 lead signals of a cohort from MADIT-CRT trial could predict the probability of survival in CRT patients more effectively than standard ECG derived dyssynchrony parameters^13^. Most recent studies^14,15^ have also shown the value of UHF ECG technology in displaying ventricular depolarization patterns during physiological and myocardial pacing.

Although HF and UHF ECG is currently being used in various clinical experiments to measure ventricular electrical activation, the validation of this technique was never performed. Furthermore, the additional value of this technique compared to standardized ECGI techniques is not yet known. Therefore, the current study aims to compare the activation patterns of heart ventricles derived from the HF ECG imaging technique (HFECGI) with epicardial activation maps derived from conventional ECGI.

## Results

### 3D HFECGI validation using ex-vivo experimental data

The aim of the experimental study is to compare the activation times determined from transmural propagation of the depolarization wave (needles), recorded epicardial potentials (sock), and the reconstructed HF potentials.

**Figure 1:**
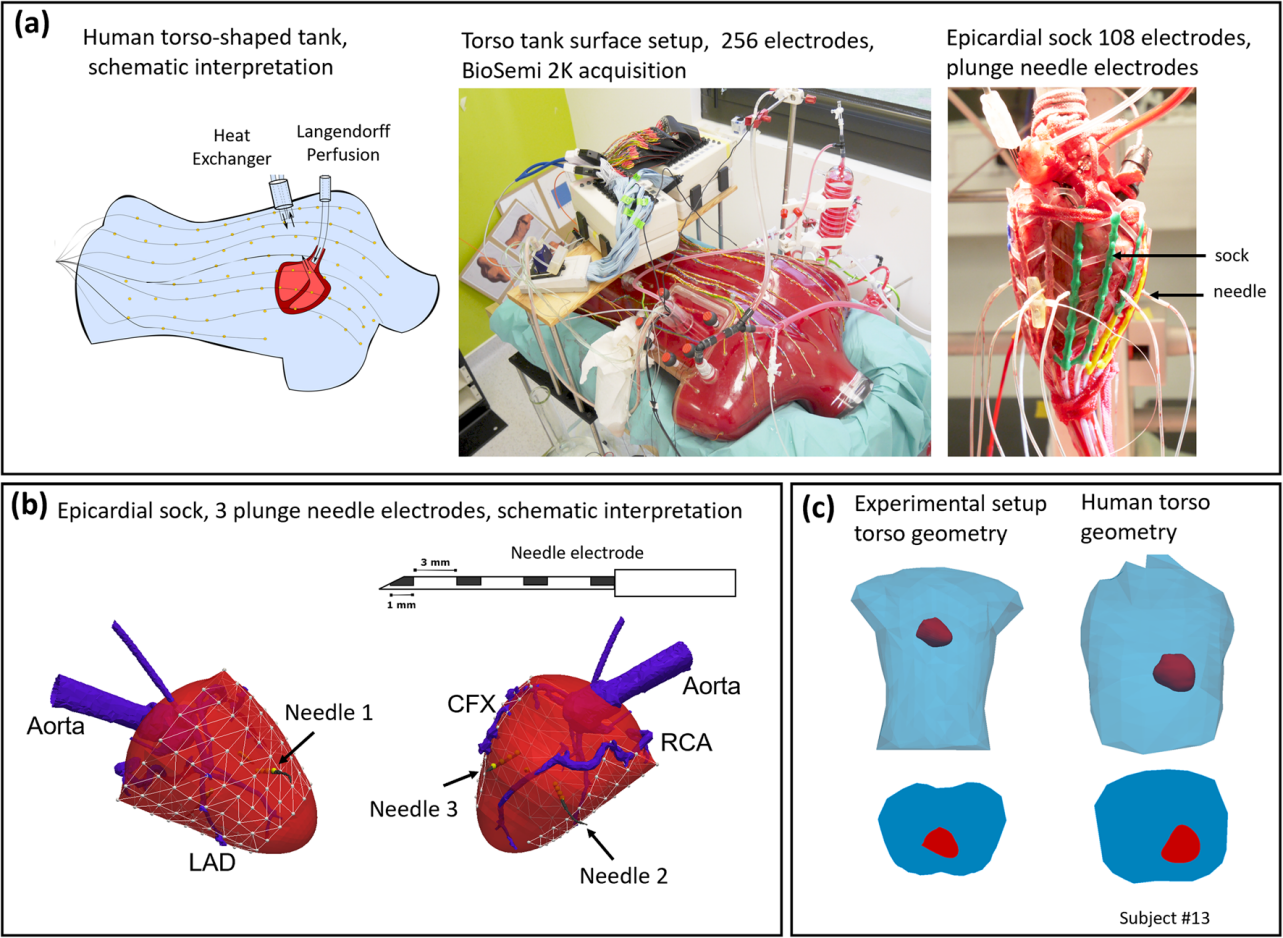
Ex-vivo experimental setup. (**a**) The Langendorff-perfused pig heart suspended in a human torso-shaped tank with 256 electrodes embedded in the torso surface and 108-electrode epicardial sock with plunge needle electrodes. (**b**) A schematic interpretation of the sock placed over the ventricle, 3 plunge electrode needles with four contacts (4 mm distance) inserted into the ventricles, LAD—left anterior descending artery, RCA—right coronary artery, CFX—circumflex artery. (**c**) Comparison of the geometry of the experimental setup with an example of the human torso.

The experimental ex-vivo setup contains two Langendorff-perfused pig hearts suspended in a human torso-shaped tank with 256 electrodes embedded in the torso surface (Fig. 1a). A detailed description of the experimental setup can be found in^7^. A 108-electrode sock was placed over both ventricles, and plunge needle electrodes with four contacts (4 mm distance) were inserted into the ventricles. Three transmural needles were used. The first needle was placed near the stimulation point; the second needle was in the right ventricle and the third one in the left ventricle. The position of the needle electrodes of the pig heart is shown in Fig. 1b. Figure 1c shows the geometry of the experimental setup and compares it with an example of a human torso.

Tank electrodes, sock potentials, and the electrical signal from the needles were simultaneously recorded with 2 kHz sampling frequency (BioSemi, The Netherlands, 0–400 Hz bandwidth, 24 bit ADC, 31 nV resolution). The same technique as for human body surface measurements was used to determine high-frequency activation times from torso recordings (HFAT). Epicardial activation times were computed directly from epicardial sock contacts (SEAT). The difference SEAT-HFAT (DIFF) indicates intramural propagation direction (positive or negative polarity).

In the two ex-vivo validation experiments, data were collected during three pacing modes. The first pig heart was paced on the left ventricular (LV) epicardial surface. The second pig heart was paced on the two locations: LV and right ventricular (RV) epicardial surface.

**Figure 2:**
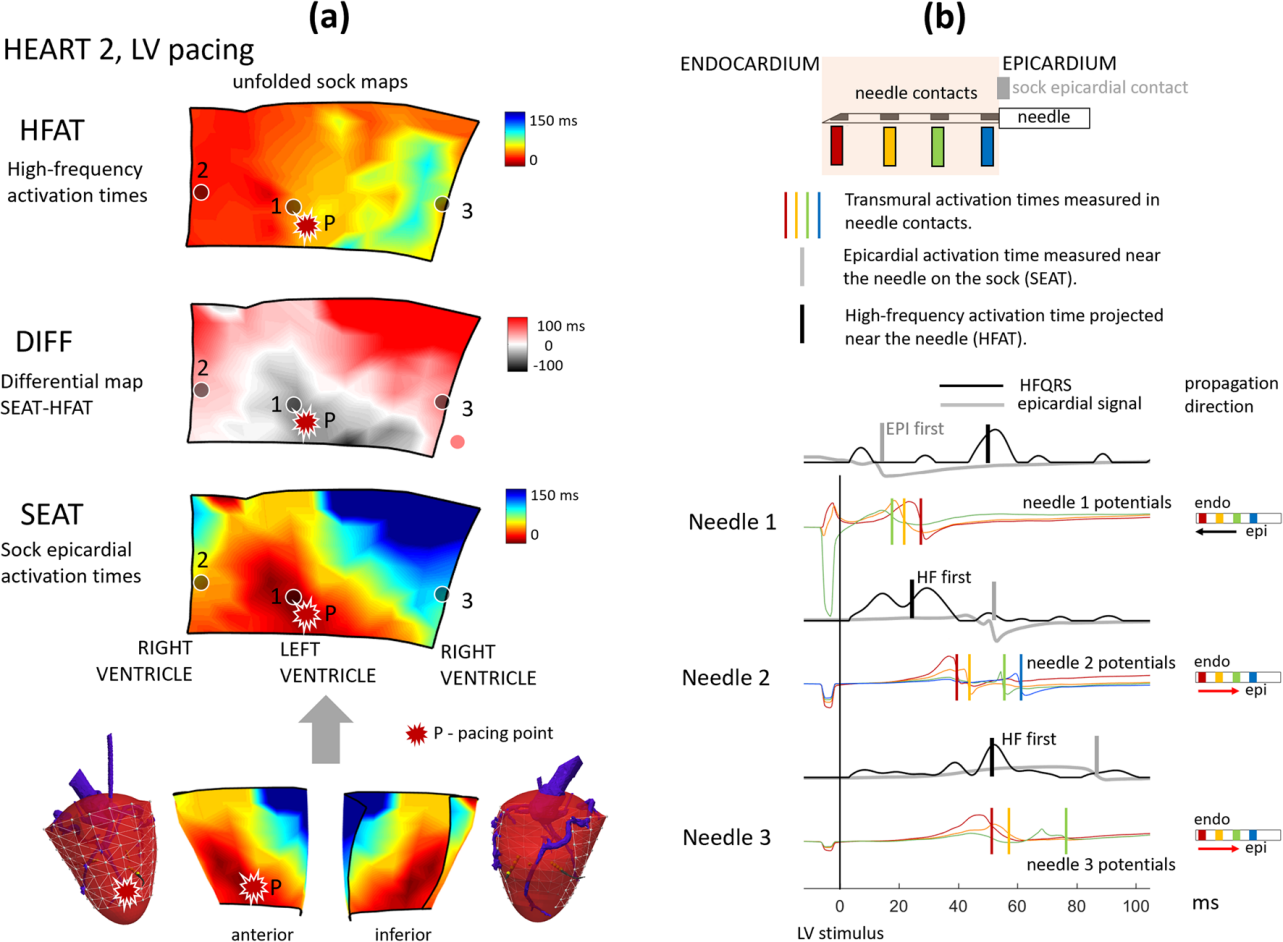
HFECGI theory validation using ex-vivo data. (**a**) Comparison of different activation maps on the sock—high-frequency activation times computed from torso records (HFAT), epicardial activation times computed from epicardial sock contacts (SEAT), and differential (DIFF) activation map computed as difference SEAT-HFAT. The red color in DIFF map means endo → epi direction, gray color means epi → endo direction (see Fig. 3). Marks 1,2,3 identify the position of needles. P marks identify the position of LV epicardial pacing. (**b**) Transmural depolarization wavefront propagation using signals from needle electrodes. Each needle electrode contained 4 contacts, marked by color (blue = close to epicardium; red = close to endocardium). The vertical color lines in the needles potentials indicate related activation time being the maximum negative derivative in each signal. If the blue contact is not shown, its position was outside the epicardial tissue. The gray signal represents epicardial potential measured on the sock contact in close proximity to the needle. The black signal represents high-frequency envelopes projected on epicardium to the needle position. The gray and black vertical marks define the epicardial activation time measured on the sock (SEAT) and the time calculated by the HFECGI method (HFAT), respectively. Complete results of two pig hearts with LV and RV pacing are shown in the Supplement, Figures S1–3.

Figure 2 and Figures S1–3 compare the shape and activation times of electrical potentials in single needle contacts and activation maps. HFAT, SEAT, and DIFF maps are shown in Fig. 2a. The independent validation of depolarization propagation direction provides the potentials measured in the needle contacts, Fig. 2b.

**Figure 3:**
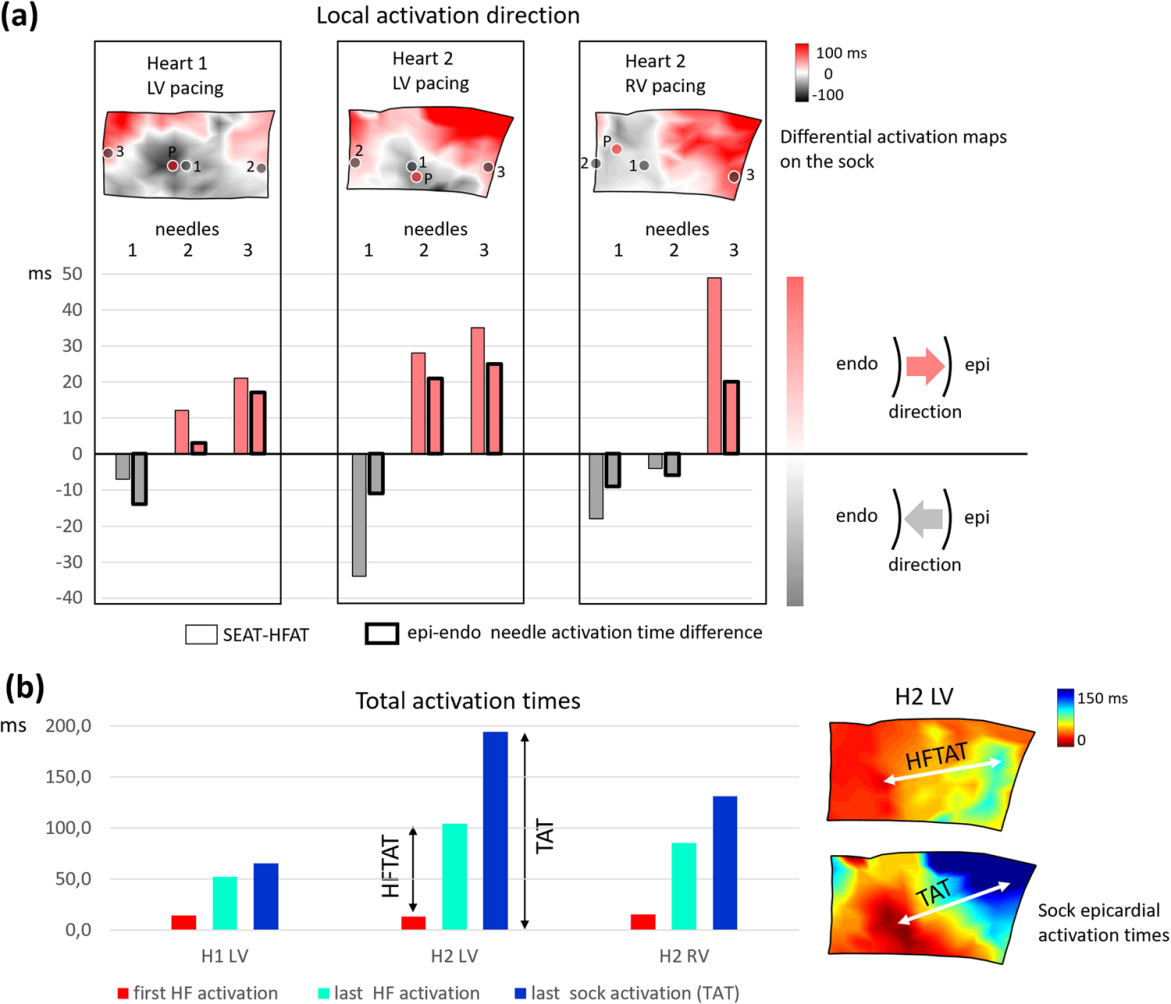
Ex-vivo comparison of the difference between epicardial sock, needle contacts, and high-frequency activation times. (**a**) Differential activation maps SEAT-HFAT, difference SEAT-HFAT near the needle (thin edges bars), and the difference between the near-epi and near-endo activation in the needle contacts (thick edges bars). Positive values indicate the direction of endo-to-epi activation (shades of red in differential maps); negative values indicate the direction of epi-to-endo activation (shades of gray in differential maps). There is a high correlation (c = 0.93) between the difference values and 100% agreement in the direction of propagation in the differential map and needle’s contacts. (**b**) Comparison of the first and the last activation in the epicardial sock map, and in the high-frequency map. The first activation time on the sock was equal to zero Thus dark blue bars (last sock activation time) define the total activation time (TAT). The difference between cyan and red bars (last and first HF activation) defines high-frequency total activation time (HFTAT).

The DIFF and the epi-endo activation difference in the needle contacts is shown in Fig. 3a. The results show a high degree of conformity between the transmural direction of depolarization in the needle electrodes and the direction that was determined from the difference between SEAT and HFAT.

Figure 3b compares high-frequency total activation time (HFTAT) with sock total activation time (TAT). It shows lower HFTAT in all three cases.

### HFECGI application in patients with different conduction abnormalities

A total of 14 patients with heart failure with reduced ejection fraction were included in the study. The characteristics of the individual patients are shown in Table S1. Six patients had left bundle branch block (LBBB), six patients had intraventricular conduction disturbance (IVCD), one patient had right bundle branch block (RBBB), and one patient had a narrow QRS complex (NORMAL) on 12-lead ECG^16^.

Body surface potentials were recorded from 184 sites around the entire surface of the human torso with BioSemi (Amsterdam, the Netherlands) hardware. The average duration of supine and resting measurement was 5 min, the sampling rate was 2 kHz, and the frequency range was up to 400 Hz. Thoracic computed tomography (CT) was performed with the electrodes attached to the patient. The body surface potentials and CT images were then processed to reconstruct patient-specific epicardial unipolar electrograms for every node (epicardial virtual points). The average number of virtual points was 2100. Inverse reconstruction of the epicardial potentials technique has previously been described and validated in an animal model^3^. The depolarization activation time (EAT) was determined as the time delay from the onset of the QRS complex to the position of the maximal negative derivative of epicardial potentials in the QRS region^3^.

**Figure 4:**
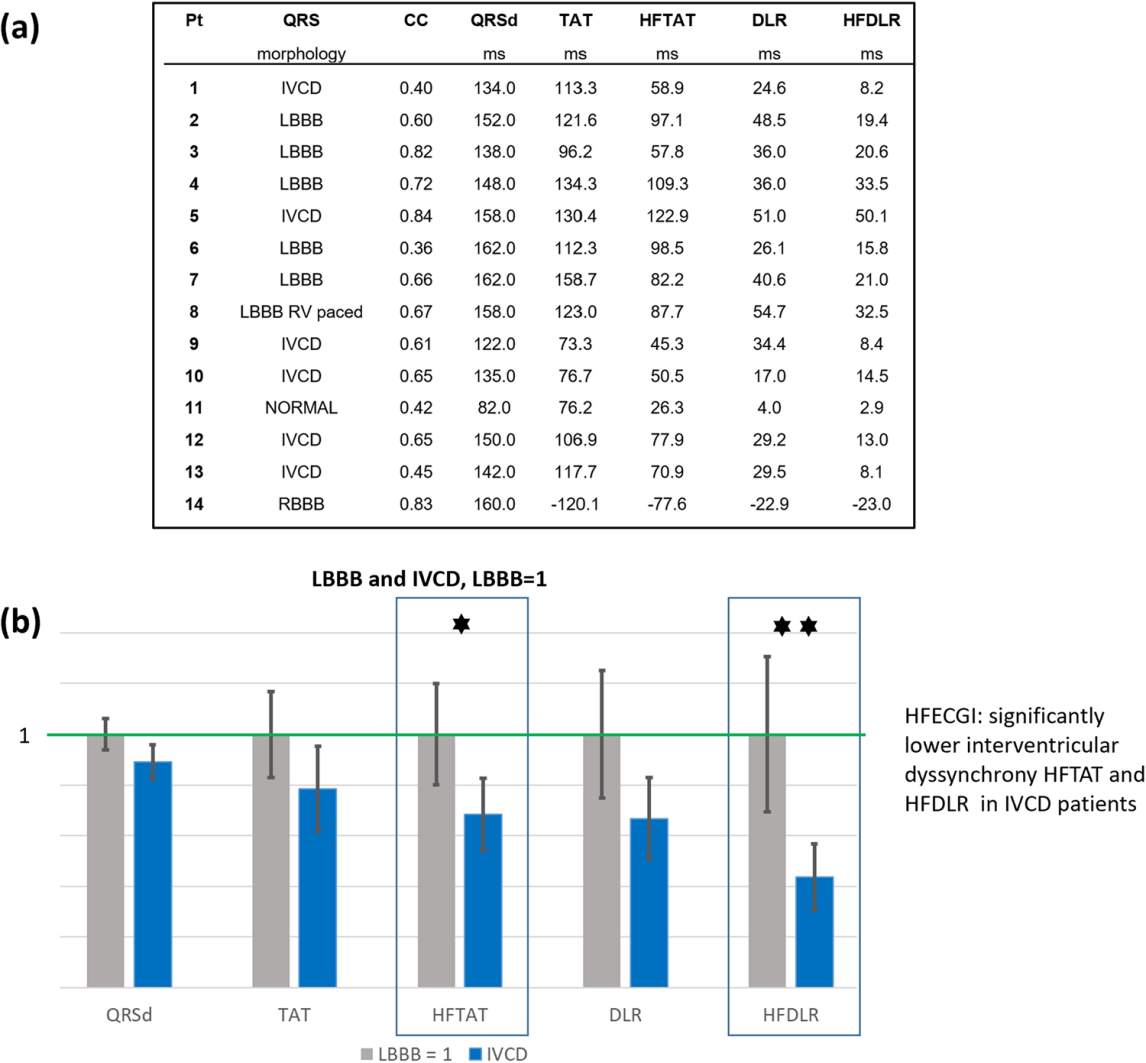
ECGI and HFECGI parameters in LBBB, IVCD, RBBB and normal patients. (**a**) Pt—patient, CC—correlation coefficient between EAT and HFAT in virtual points, QRSd—QRS duration, TAT, HFTAT—total activation time, DLR, HFDLR—interventricular electrical delay. (**b**) Relative magnitudes of parameters in IVCD patients compared to LBBB patients (mean LBBB = 1), *p < 0.05, **p < 0.01. HFECGI parameters HFTAT and HFDLR indicate significantly lower dyssynchrony in IVCD patients. Mean values and standard deviation.

**Figure 5:**
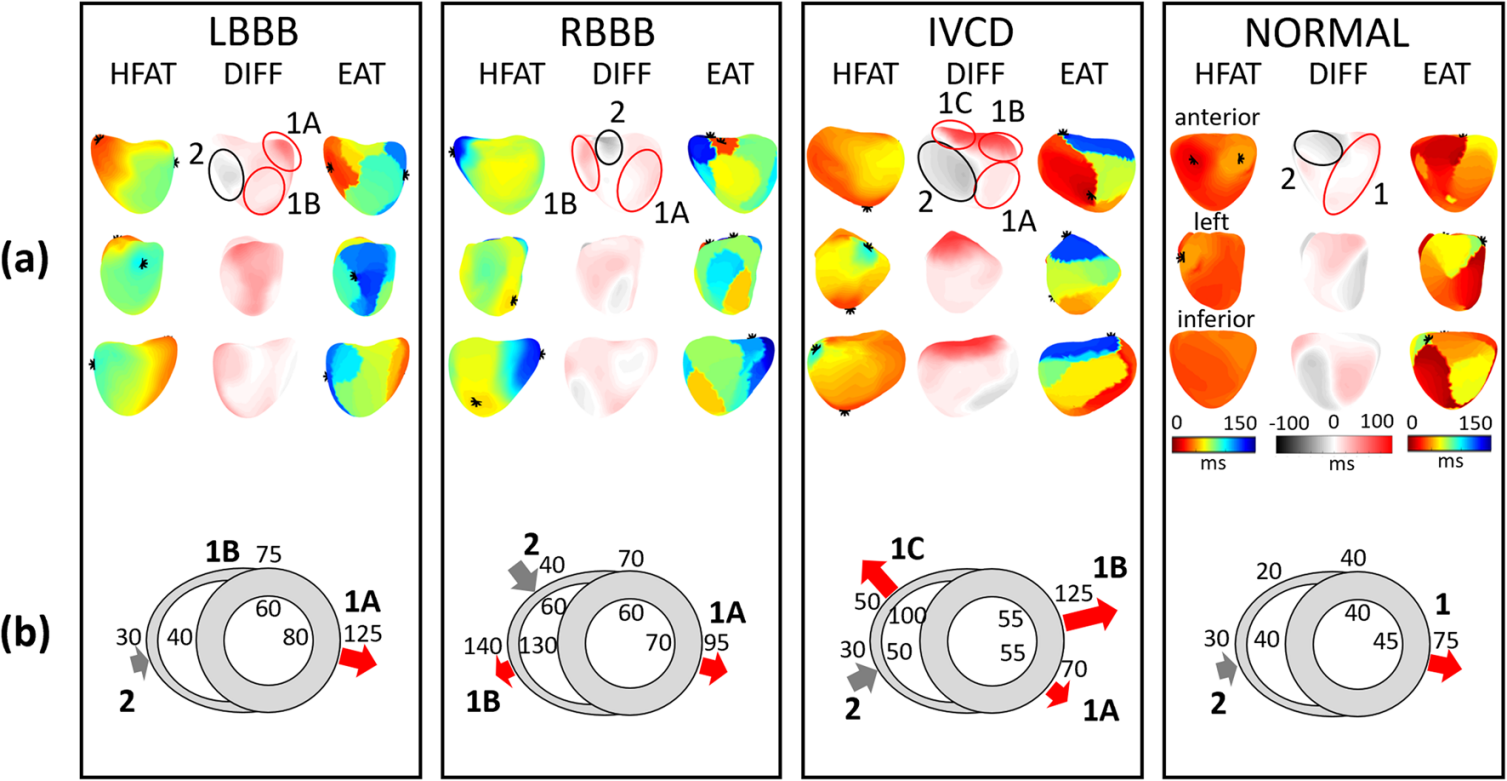
Three-dimensional activation pattern interpretation. High-frequency activation times (HFAT), epicardial activation times (EAT), and differences (DIFF) in four exemplary patients: Left bundle branch block (LBBB, patient #3), right bundle branch block (RBBB, patient #14), intraventricular conduction disturbance (IVCD, patient #1) and normal synchronous heart (NORMAL, patient #11). (**a**) HFAT, DIFF, and EAT maps. Each map is shown from three projections (anterior, left, and inferior). (**b**) The red and gray arrows indicate the direction of electrical activation propagation derived from the EAT-HFAT plot in the second row; the size of the arrow corresponds to the endo-epi time delay. Numbers show the approximate timing of electrical activation.

Numerical results of all patients and the differences between the LBBB and IVCD groups are shown in Fig. 4. Figure 5 shows HFAT, DIFF, and EAT maps and schematic interpretation of transmural electrical activation propagation in four exemplary subjects with LBBB, RBBB, IVCD, and narrow QRS complex. HFAT, DIFF, and EAT maps, including scatter plots of all patients, are shown in the Supplement, Figures S4–6.

The activation maps show activation sequences that are characteristic of these types of patients. The mean correlation coefficient between EAT and HFAT maps for all subjects was 0.66; (0.69 for LBBB, 0.60 for IVCD, 0.83 for RBBB, and 0.42 for the patient with narrow QRS complex).

Dyssynchrony parameters HFAT and high-frequency interventricular electrical delay (HFDLR) were significantly smaller than their EAT counterparts (p < 0.001). Over all subjects, TAT and HFAT represented 78 and 52% of QRS width, respectively. In absolute values, HFTAT was approximately 40 ms lower than TAT. HFAT was minimal (only 26 ms) in the normal heart. HFTAT and HFDLR showed significantly smaller dyssynchrony in IVCD patients in comparison with LBBB patients, while the EAT dyssynchrony measures were not significantly different (Fig. 4b).

Figure 5 illustrates the interpretation of the transmural activation patterns. Endo-epi propagation dominates in most regions, as shown by the red arrows, except for areas of early activation in all four cases (area 2). This behavior corresponds to expected physiological activation. Of particular interest are the regional differences in transmural activation within the RV of the RBBB and IVCD patients.

## Discussion

The experimental results confirmed two outcomes presented in Figs. 2 and 3: (1) High-frequency dyssynchrony expressed by HFTAT was lower than epicardial TAT dyssynchrony. It indicates that HFAT measures averaged transmural activation. (2) The sign of the difference between SEAT and HFAT (direction of depolarization propagation) correlates with transmural signal propagation direction in needles. It confirms that the difference between EAT and HFAT can identify the propagation direction of the depolarization wave. This evidence provided by ex-vivo experiments confirms the relationship between clinical EAT and HFAT.

Because of the extensive technical requirements, only a few reports provide detailed information about transmural activation in the ventricles^17,18^. Durrer et al. used multiple transmural needles to collect data from approximately 700 electrodes in isolated perfused explanted human hearts^19^. Similar measurements, including ventricular pacing, were performed in dog hearts^20^. Nanthakumar et al.^21^ used an epicardial sock and endocardial balloon to determine activation in patients during open-heart surgery. This approach was also used in a canine LBBB model^22^. Clinically, extensive invasive combined endocardial and epicardial mapping is sometimes used, for example, to map and ablate the focus of ectopic activation^23^. This hybrid approach is usually only done after a failed endocardial approach in very limited cases. Therefore, the ability to noninvasively acquire information about transmural activation patterns in patients is unique. The only approach that may lead to similar information is the combination of ECG measurements and computer modeling^24^. Like our method, this approach still requires further validation.

Various observations support the reliability of the transmural conduction in our measurements. (1) The lower HFTAT than EAT values, indicating smaller electrical delays between midwalls than between the epicardium of opposing walls, are logical because the latest activated region is in the epicardium. (2) The mean difference between HFAT and EAT is smaller in the RV than in the LV, which is probably attributable to the difference in wall thickness between the ventricles. (3) Transmural differences across the LV are in the range of those observed during direct measurements^19,20,22^. (4) The very low HFTAT value in the patient with a narrow QRS complex is a logical physiological measure in a heart with a normal conduction system, ventricular activation occurs predominantly from the endocardium to the epicardium. The ventricular volume is activated rapidly, and this corresponds to a low HFTAT value^19^.

An interesting and important observation in this study was that the HFECGI parameter HFDLR showed a significant difference between LBBB and IVCD patients, whereas this was not the case for its ECGI-derived counterpart interventricular electrical delay (DLR). This observation may be explained by the fact that in IVCD, the transmural conduction plays a more important role because the Purkinje system is likely (more) intact^23^. Indeed, the EAT-HFAT plots of IVCD patients show a very flat slope with more points below the line of unity in the LV, indicating a large transmural activation difference (see Supplement, Figure S6, IVCD). We speculate that such better insight into the transmural activation sequence may become an additional tool to discriminate between LBBB and IVCD, enhancing patient selection for CRT.

The additional information provided by HFECGI and its combination with ECGI can be obtained with relatively little effort: data collection for at least one minute and at the high sampling frequency. While recording times are shorter in some ECGI applications, others already use ECG averaging. Averaging is required in HFECGI to obtain reliable signals due to the low amplitudes in the higher frequency domains. The frequency content of a conventional ECGI device is often limited to 250 Hz (sampling rate max 1 kHz). However, increasing the bandwidth of the ECG recorders is not a significant problem in technical terms.

EAT (potential-based inverse ECGI) maps show several activation time “jumps”. Recently, it was shown that such jumps are most likely an artifact^25,26^. For this reason, we use smoothed EAT maps. Each virtual point is computed as an average from 6 surrounding virtual points. This problem does not occur with HFECGI.

### Translational outlook

The clinical impact may well go beyond the improved investigation of ventricular conduction abnormalities. After all, on many occasions, it is important to know the transmural course of activation. By assessing epicardial and transmural activation during different pacing settings, one may better understand the physiology and pathophysiology of failed resynchronization and opt for alternative resynchronization approaches such as His bundle pacing, LV septum pacing, or endocardial LV pacing^12,14,15^.

The utility of HFECGI additionally may be useful in patients with symptomatic ventricular ectopic beats and refractory ventricular tachycardia to decide whether an endocardial or epicardial approach should be applied.

This study explains the HF ECG mechanism to determine dyssynchronous activation and its validation with the ex-vivo approach. The HF ECG theory will help better understand the activation maps obtained by HF and UHF 14-lead ECG technology recently introduced^12,14,27,28^.

*Limitations* (1) The number of experiments and patients limits the interpretation of the results. Additional ex-vivo experiments and more transmural needles will be needed to determine details in ECGI and HFECGI dependencies accurately. However, the basic principles have been formulated and discussed. (2) The study is based on ECG data with a limited bandwidth of up to 400 Hz. In 12–14-lead ECG based ventricular activation mapping^12^, the UHF bandwidth up to 1000 Hz is used. The broader frequency band allows higher averaging in the frequency domain and provides more accurate results.

## Conclusions

This study introduces the HFECGI technique, its interpretation, and the basic principles of high-frequency electrocardiography. HFAT defines transmural volume activation, and clinical data indicate that HFAT results may differentiate between LBBB and IVCD patients. We also demonstrate that the combination of EAT and HFAT activation maps may provide a more detailed view of ventricular electrical activation propagation.

## Methods

### High-frequency activation time (HFAT) computation

**Figure 6:**
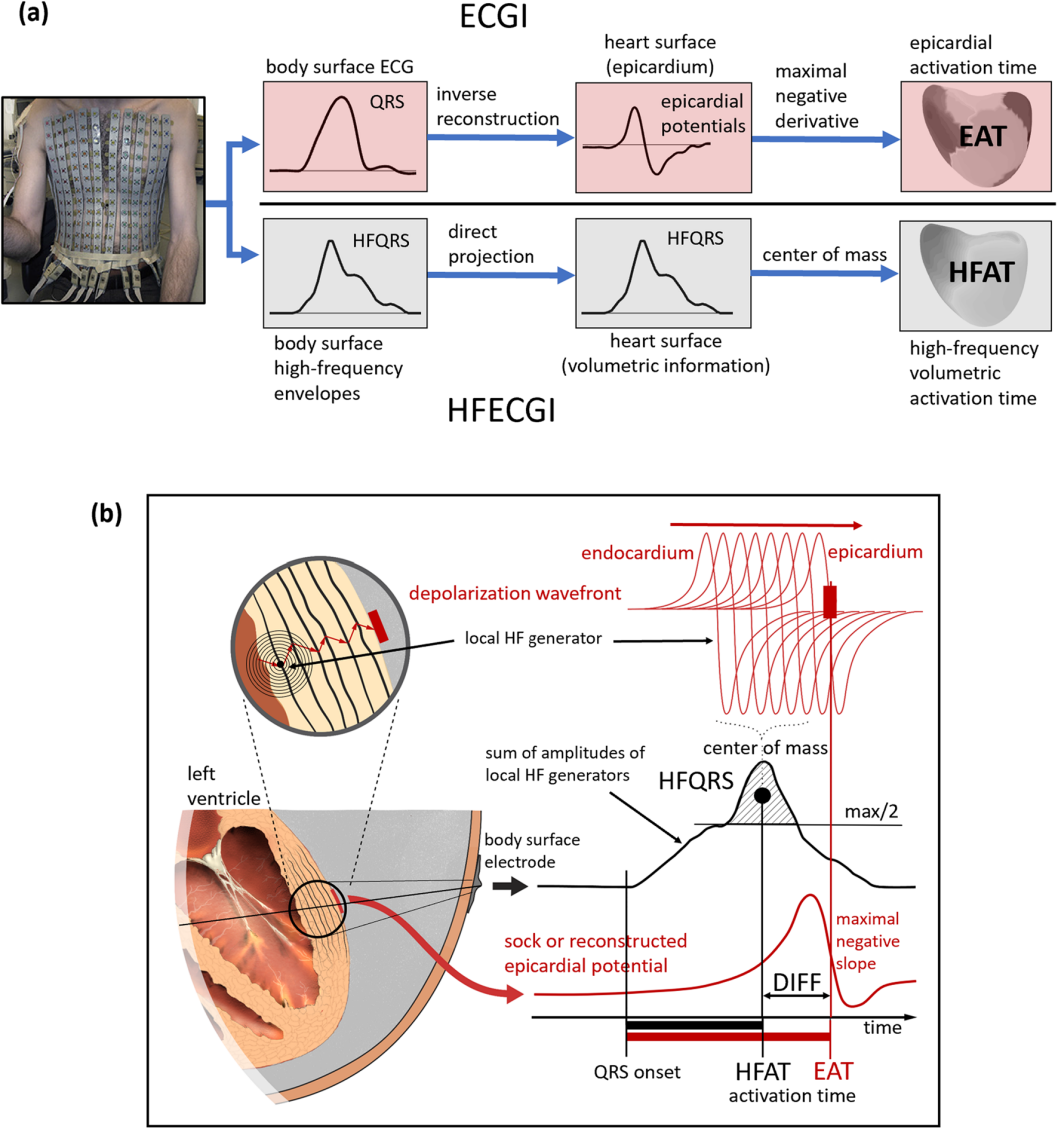
High-frequency electrocardiographic imaging. (**a**) Block diagram that compares the electrocardiographic imaging (ECGI) technique and high-frequency (HF) electrocardiographic imaging (HFECGI) technique. (**b**) Estimation and interpretation of epicardial activation time (EAT), HF activation time (HFAT), and their difference (DIFF). EAT is computed as the delay between the QRS onset and the maximal negative slope of epicardial potential. It corresponds to the time when the depolarization wave reaches the epicardium. HFAT is defined as the delay between the QRS onset and the center of mass (hatched area) computed from HFQRS projected on the epicardium. In this way, HFAT reflects the time when the largest amount of myocardial cells is depolarized simultaneously—activation at approximately halfway on the depolarization path across the wall. DIFF then indicates the delay between the midwall and epicardial activation.

Figure 6 shows the principle of HFECGI, the method of HFAT determination, and the difference between HFECGI and ECGI.

QRSs from precordial V1–V6 leads were used for the detection of regular and irregular heartbeats. A robust multichannel approach was used to select the regular (dominant) beats^29^ for further processing. The amplitude envelopes of each torso or body surface potential (referenced to Wilson's central terminal) were computed in the five frequency bands 100–200, 150–250, 200–300, 250–350, and 300–400 Hz using the Hilbert transform. In each frequency band, the averaged QRS amplitude envelopes were computed with an R-wave trigger. The high-frequency body surface QRS complex (HFQRS) was then constructed as the mean of the normalized averaged envelopes over all frequency bands. Averaging across frequency bands increases the signal-to-noise ratio and stabilizes the result. The frequency averaging and its effect on HF and UHF amplitude envelope is discussed in Figure S8 and described in detail in^12^.

The body surface HFQRS was projected onto the epicardium, using the same virtual points which were created for the inverse ECGI reconstruction. The direct projection from the surface of the body to the epicardium using the geometric center of the heart was used (Fig. 6b). A detailed description of the direct projection is in Figure S7. The HF activation time HFAT of a single virtual point on the epicardium was determined as the time delay from the onset of the QRS complex to the center of mass of the HFQRS. The HFQRS center of mass can be interpreted as a time when the maximal amount of myocardial cells is activated simultaneously. The center of mass corresponds better to the volumetric activation time than the maximum. We can also assume that the thus determined activation time of electrical depolarization corresponds more closely to the subsequent mechanical contraction.

### Numerical parameters

Electrical dyssynchrony related parameters were determined for each patient:

- total activation time—**TAT**, the difference between the first and the last EAT activation on epicardium (epicardial ventricular electrical dyssynchrony);
- high-frequency total activation time—**HFTAT**, the difference between the first and the last HFAT activation in transmural wall sections;
- interventricular electrical delay—**DLR**, the difference between the mean EAT activation time of LV and RV free walls (LVFW, RVFW);
- high-frequency interventricular electrical delay—**HFDLR**, the difference between the mean HFAT activation time of LVFW and RVFW. Positive DLR and HFDLR values reflecting delayed LV activation, negative values delayed RV activation.

### Three-dimensional (3D) electrical activation pattern

A combination of a low-frequency (LF) ECGI and high-frequency (HF) HFECGI results provides information that allows to interpret a 3D electrical activation pattern. Both LF and HF components are contained in all HF ECG records and are processed simultaneously.

The LF and HF components have different origins. Body surface LF ECG is based on the lead-field projection of electrical vectors perpendicular to the depolarization wavefront. Microscopic delays in conduction and fast change in current and voltage during phase 0 of myocardial cells depolarization action potentials lead to HF components. The LF components have a vector character while the HF components propagate like spherical waves. Thanks to amplitude decrease with the distance from the source, it is not possible to identify different HF sources on the limb leads, and it is possible to analyze them only in the close area around the heart on the chest.

**Figure 7:**
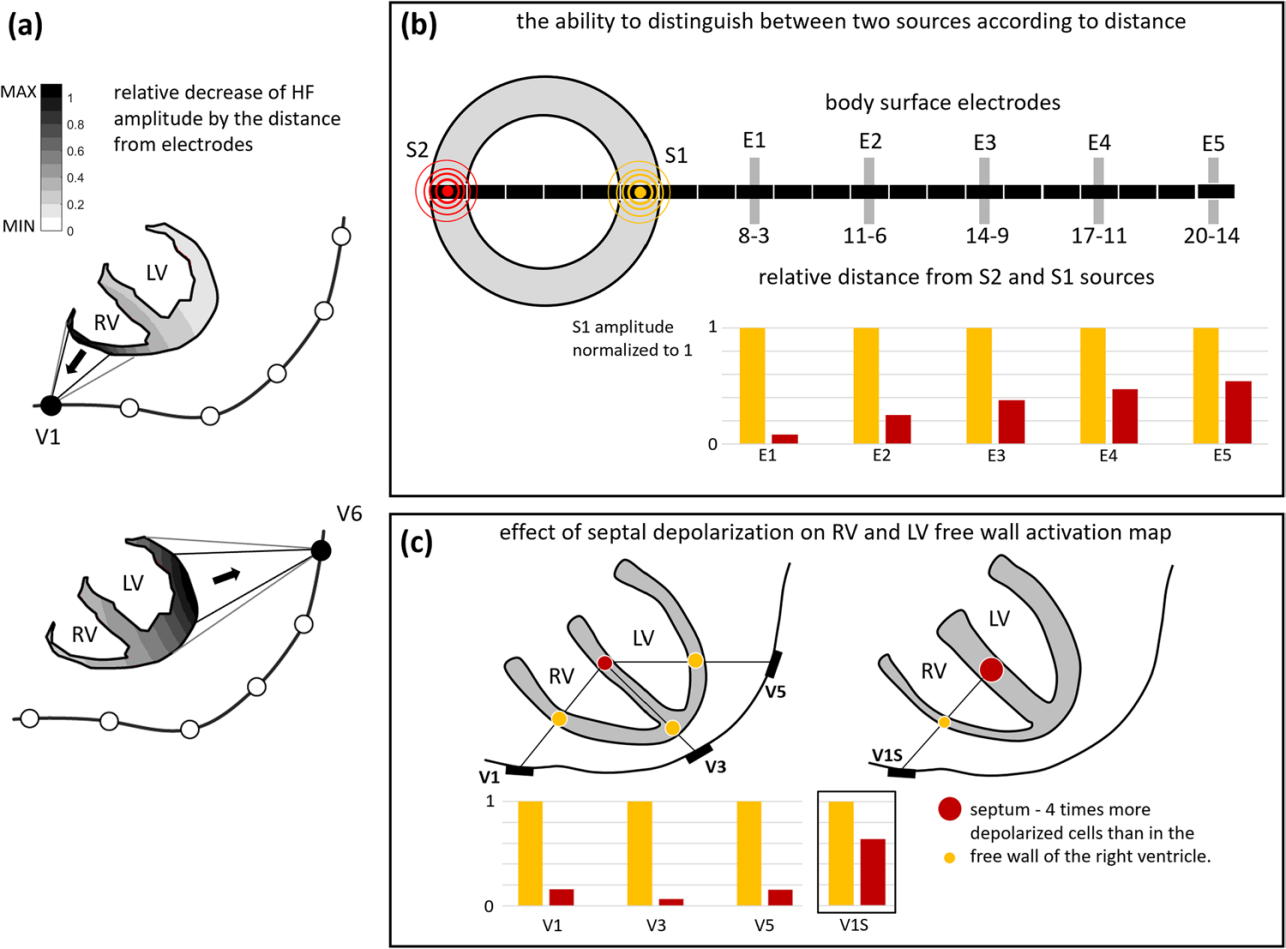
High-frequency ECG spatial sensitivity. The HF signal amplitude at a given time is the sum of simultaneously depolarized myocardial cells. Single HF source contribution is determined by the distance of the myocardial cell from the body surface electrode. The contribution decreases approximately with the square of the distance. (**a**) The black color shows areas with the largest contribution, light grey with the lowest contribution. For a given geometry, in the case of the V1 electrode, the right ventricular signal is 10 times stronger than the left ventricular signal. In the case of the V6 electrode, the right ventricular signal contribution is approximately 5 times weaker than the left ventricular signal (free wall) contribution. (**b**) Comparison of the measured HF amplitude of two different equivalent sources (S1, S2) in five body surface electrodes with different distances (E1-5). The closer the electrode is to the sources (E1), the better it can distinguish them—a higher difference between the amplitude of the near and far source. (**c**) Example of the mid septum activation (red circle) contribution to V1 (RV), V3 (apex), and V5 (LV free wall) electrodes. The lowest mid septum activation effect is in the V3 electrode—because V3 is closest to the source from the apex. The number of simultaneously activated myocardial cells proportionally increases the amplitude of HF oscillations. The area of the depolarization wavefront is larger in the septum than in the thin free wall of the RV. Here is an example of a four times higher amount of simultaneously depolarized myocardial cells in the septum (right, signal V1S). If the RV and septum are activated at the same time, both sources are added. If the septum is activated first and later the RV free wall (RBBB), the HF amplitude shows bifurcation—two peaks.

HF components do not reflect the depolarization propagation direction along or across the wall. Only a concurrent analysis of HFECGI and ECGI provides this information. The result of ECGI are reconstructed LF potentials on the epicardium. Each virtual point on epicardium is an inverse transformation of all body surface potentials. The HFECGI computes the HF signal amplitude in all body surface ECG electrodes and uses direct projection to a virtual point on the epicardium. Single lead HF amplitude reflects the activation in the myocardial wall close to the electrode (Fig. 6b). HFECGI spatial sensitivity is demonstrated in Fig. 7. The closer the body surface electrode is to the different sources, the better it can distinguish their dyssynchronous activation. In such a case, the amplitude of the remote source is substantially lower than the amplitude of the nearby source.

Figure 7c also shows how septal electrical activation affects HFECGI activation maps. The activated septum minimally affects the apex area and the free wall of the left ventricle. This is because the activated volumes are comparable, and the septum is significantly distant from the body surface electrodes (V3, V5). The situation is different in the case of a free wall of the right ventricle where the activation volumes are lower than the septal. In RV, it is necessary to take into account that HFECGI maps also reflect the activation of the septum (V1S).

Distant leads or limb leads cannot distinguish sources. In addition, with the distance increase, the amplitude of the HF oscillations and the signal-to-noise ratio decrease. Therefore, thoracic electrodes close to the heart ventricles have crucial significance for the diagnosis of ventricular dyssynchrony.

**Figure 8:**
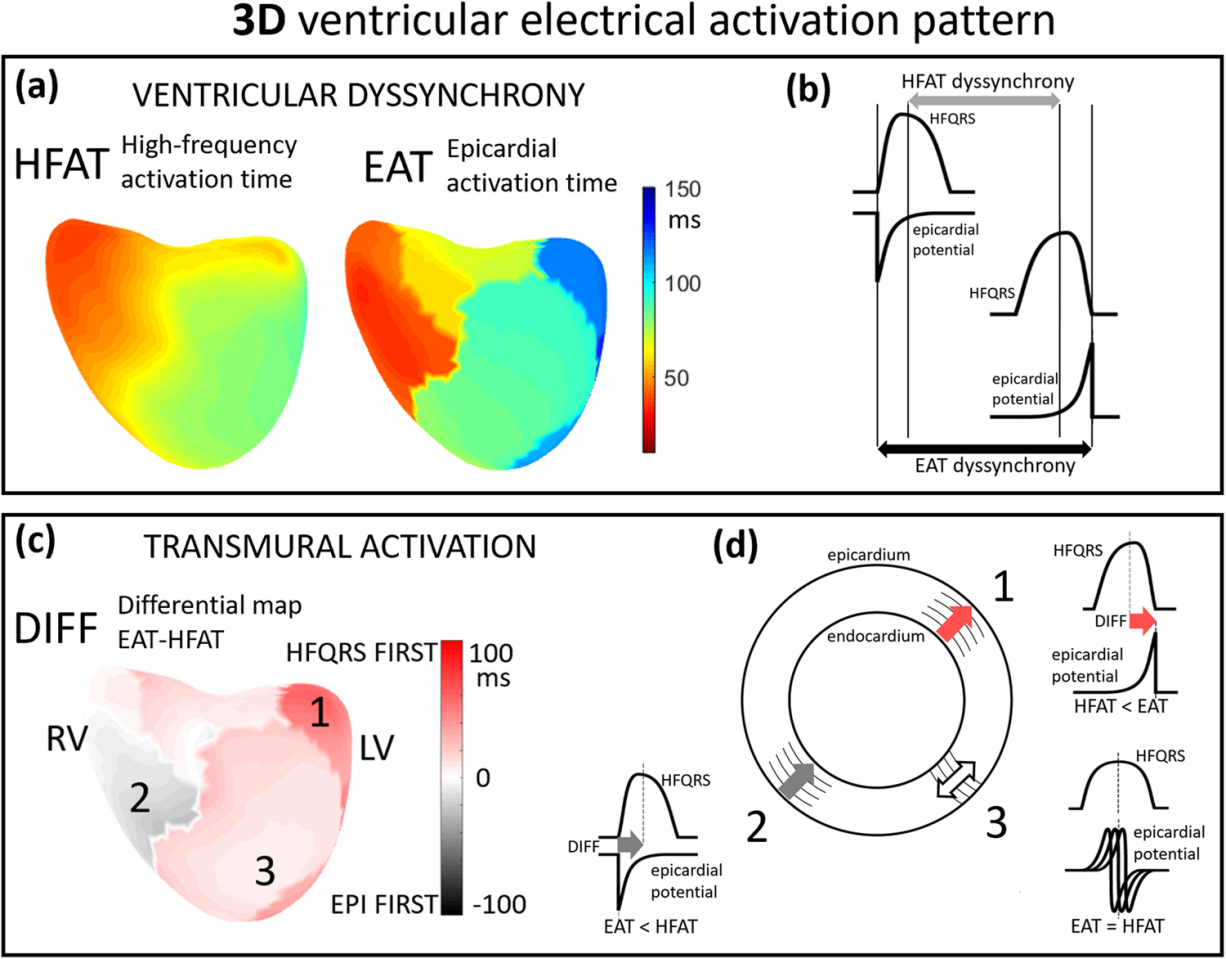
Interpretation of difference between ECGI and HFECGI-derived activation times. (**a**) High-frequency activation time (HFAT) and epicardial activation time (EAT) maps (anterior view) of patient #3 (LBBB). The red color indicates earlier activation, and the blue color indicates later activation. (**b**) Schematic representation of ventricular dyssynchrony based on EAT and HFAT. (**c**) DIFF map; red tones indicate EAT > HFAT and gray tones HFAT > EAT. (**d**) Interpretation of DIFF values. In area 1 HFAT is smaller (sooner) than EAT (marked by red arrow), representing a dominant propagation directed from the endocardium to the epicardium. In area 2 the prevailing direction of propagation is opposite because EAT precedes HFAT (marked by the gray arrow). In areas with similar EAT and HFAT values (area 3), the dominant direction of propagation is along the wall, or there are turbulences in depolarization propagation.

The 3D features of the combination of both techniques are shown in Fig. 8. Because the time of maximal negative slope of epicardial potentials (EAT) corresponds to the time of epicardial activation and HFAT to the transmural activation, a difference between EAT and HFAT at a specific location reflects the duration and direction of propagation of the depolarization wave. The difference (DIFF) between EAT and HFAT provides information about the direction of wavefront propagation. If EAT < HFAT, conduction occurs from the epicardium to the endocardium, whereas if HFAT < EAT, conduction occurs from the endocardium to the epicardium. DIFF values near zero indicate no dominant endo-to-epi or epi-to-endo electrical wavefront propagation.

### Statistical analysis

Statistical analyses were performed in MATLAB 2018B (The MathWorks, Inc., Massachusetts, USA). The linear Pearson correlation coefficient CC between EAT and HFAT activation times was computed over all virtual points for each patient. Statistical comparison of EAT and HFAT markers was performed by a Wilcoxson signed-rank test for paired measurements. Scatter plots and histograms (Supplement, Figures S5, S6) of the time distribution of the activation times of virtual points were used for graphical interpretation of EAT and HFAT activation time relationship and differences.

### Approval for clinical and animal experiments

*Clinical experiments* All methods were carried out in accordance with relevant guidelines and regulations. The clinical study was approved by the Medical Ethics Committee of Maastricht University Medical Center. Patients gave written consent to participation in the study and the use of the data.

*Ex-vivo experiments* All experiments were performed in accordance with relevant guidelines and regulations. Hearts were excised from pigs (n = 2, 30–40 kg) as approved by Directive 2010/63/EU of the European Parliament on the protection of animals used for scientific purposes and the local ethic committee of Bordeaux CEEA50.

## Supplementary Information

Supplementary Information.

## Data Availability

We comply with the Scientific reports data availability policy, and we will make any data available to reviewers or referees if needed.

## References

[1] Cluitmans M (2018). Validation and opportunities of electrocardiographic imaging: From technical achievements to clinical applications. Front. Physiol..

[2] Oster HS, Taccardi B, Lux RL, Ershler PR, Rudy Y (1998). Electrocardiographic imaging: Noninvasive characterization of intramural myocardial activation from inverse-reconstructed epicardial potentials and electrograms. Circulation.

[3] Cluitmans MJM (2017). In vivo validation of electrocardiographic imaging. JACC Clin. Electrophysiol..

[4] Bear LR (2018). How accurate is inverse electrocardiographic mapping?. Circ. Arrhythm. Electrophysiol..

[5] Sapp JL, Dawoud F, Clements JC, Horácek BM (2012). Inverse solution mapping of epicardial potentials: Quantitative comparison with epicardial contact mapping. Circ. Arrhythm. Electrophysiol..

[6] Ramanathan C, Ghanem RN, Jia P, Ryu K, Rudy Y (2004). Noninvasive electrocardiographic imaging for cardiac electrophysiology and arrhythmia. Nat. Med..

[7] Bear LR (2018). Cardiac electrical dyssynchrony is accurately detected by noninvasive electrocardiographic imaging. Heart Rhythm.

[8] Ploux S (2013). Noninvasive electrocardiographic mapping to improve patient selection for cardiac resynchronization therapy. J. Am. Coll. Cardiol..

[9] Nguyên UC (2017). Evaluation of the use of unipolar voltage amplitudes for detection of myocardial scar assessed by cardiac magnetic resonance imaging in heart failure patients. PLoS ONE.

[10] Nguyên UC, Mafi-Rad M, Aben J-P, Smulders MW, Engels EB, van Stipdonk AMW (2017). A novel approach for left ventricular lead placement in cardiac resynchronization therapy: Intraprocedural integration of coronary venous electroanatomic mapping with delayed enhancement cardiac magnetic resonance imaging. Heart Rhythm.

[11] Jurak P (2017). Ventricular dyssynchrony assessment using ultra-high frequency ECG technique. J. Interv. Card. Electrophysiol..

[12] Jurak P (2020). Novel ultra-high-frequency electrocardiogram tool for the description of the ventricular depolarization pattern before and during cardiac resynchronization. J. Cardiovasc. Electrophysiol..

[13] Plesinger F (2018). Ventricular electrical delay measured from body surface ECGs is associated with cardiac resynchronization therapy response in left bundle branch block patients from the MADIT-CRT Trial (Multicenter Automatic Defibrillator Implantation-Cardiac Resynchronization Therapy). Circ. Arrhythm. Electrophysiol..

[14] Curila K (2020). Both selective and nonselective his bundle, but not myocardial, pacing preserve ventricular electrical synchrony assessed by ultra-high-frequency ECG. Heart Rhythm.

[15] Curila K (2021). Ventricular activation pattern assessment during right ventricular pacing: Ultra-high-frequency ECG study. J. Cardiovasc. Electrophysiol..

[16] Nguyên UC (2018). Integration of cardiac magnetic resonance imaging, electrocardiographic imaging, and coronary venous computed tomography angiography for guidance of left ventricular lead positioning. Europace.

[17] Opthof T (2017). Cardiac activation–repolarization patterns and ion channel expression mapping in intact isolated normal human hearts. Heart Rhythm.

[18] Caldwell BJ (2009). Three distinct directions of intramural activation reveal nonuniform side-to-side electrical coupling of ventricular myocytes. Circ. Arrhythm. Electrophysiol..

[19] Durrer D (1970). Total excitation of the isolated human heart. Circulation.

[20] Taccardi B, Punske BB, Macchi E, Macleod RS, Ershler PR (2008). Epicardial and intramural excitation during ventricular pacing: Effect of myocardial structure. Am. J. Physiol. Heart. Circ. Physiol..

[21] Nanthakumar K (2010). Intraoperative high-density global mapping in adult-repaired tetralogy of fallot altered left ventricular and right ventricular activation and implications for resynchronization strategies. J. Am. Coll. Cardiol..

[22] Strik M (2013). Interplay of electrical wavefronts as determinant of the response to cardiac resynchronization therapy in dyssynchronous canine hearts. Circ. Arrhythm. Electrophysiol..

[23] Derval N (2017). Distinctive left ventricular activations associated with ECG pattern in heart failure patients. Circ. Arrhythm. Electrophysiol..

[24] van Dam PM (2017). Electrocardiographic imaging-based recognition of possible induced bundle branch blocks during transcatheter aortic valve implantations. Europace.

[25] Duchateau J (2018). Performance and limitations of noninvasive cardiac activation mapping. Heart Rhythm.

[26] Duchateau J, Potse M, Dubois R (2017). Spatially coherent activation maps for electrocardiographic imaging. IEEE Trans. Biomed. Eng..

[27] Halamek J (2019). The relationship between ECG predictors of cardiac resynchronization therapy benefit. PLoS ONE.

[28] Leinveber P (2016). The relationship between mechanical and electrical dyssynchrony. Comput. Cardiol..

[29] Plesinger F (2017). The VED meter—A new tool to measure the ventricular conduction abnormalities in heart failure patients. Comput. Cardiol..

