# Supplementary material for: 3-Dimensional ventricular electrical activation pattern assessed from a novel high-frequency electrocardiographic imaging technique: principles and clinical importance
Source: Sci Rep. 2021 Jun 1;11:11469. doi: 10.1038/s41598-021-90963-4 (PMC8169848; doi:10.1038/s41598-021-90963-4)
Supplement: Supplementary file 1 — Supplementary Information. [file 41598_2021_90963_MOESM1_ESM.docx]

**Supplementary materials**

**3-Dimensional Ventricular Electrical Activation Pattern Assessed from a Novel** **High-Frequency Electrocardiographic Imaging Technique: Principles and Clinical Importance**

Pavel Jurak, Laura R. Bear, Uyên Châu Nguyên, Ivo Viscor, Petr Andrla, Filip Plesinger, Josef Halamek, Vlastimil Vondra, Emma Abell, Matthijs J. M. Cluitmans, Rémi Dubois, Karol Curila, Pavel Leinveber, Frits W. Prinzen

**Results**

***Table S1. Patient characteristics***

| **Pt** | **Sex** | **Age** | **iCMP** | **NYHA** | **DE-CMR*** | **LVEF** | **ECG rhythm** | **QRS morphology** |
| --- | --- | --- | --- | --- | --- | --- | --- | --- |
| 1 | M | 70 | Yes | 2 | 13% | 28% | AF | IVCD |
| 2 | M | 67 | Yes | 2 | 21% | 28% | Sinus | LBBB |
| 3 | M | 72 | Yes | 2 | 15% | 11% | Sinus | LBBB |
| 4 | F | 77 | No | 2 | 0% | 25% | Sinus | LBBB |
| 5 | F | 59 | Yes | 3 | 32% | 20% | Sinus | IVCD |
| 6 | M | 70 | Yes | 2 | 28% | 36% | Sinus | LBBB |
| 7 | M | 79 | Yes | 3 | No CMR | 30% | RVpaced | LBBB |
| 8 | M | 66 | No | 2 | 0% | 24% | Sinus | LBBB |
| 9 | M | 80 | No | 3 | 31% | 22% | Sinus | IVCD |
| 10 | M | 76 | Yes | 3 | 10% | 25% | AF | IVCD |
| 11 | F | 53 | No | 3 | 0% | 23% | Sinus | Narrow |
| 12 | M | 70 | Yes | 2 | 28% | 10% | Sinus | IVCD |
| 13 | M | 80 | Yes | 2 | 15% | 20% | AF | IVCD |
| 14 | M | 72 | Yes | 2 | 38% | 23% | Sinus | RBBB |

*Pt – patient, AF – atrial fibrillation, F – female, iCMP – ischemic cardiomyopathy, DE-CMR - gadolinium contrast enhanced cardiac magnetic resonance imaging, No CMR - CMR was not performed, IVCD – intraventricular conduction disturbance, LBBB – left bundle branch block, LVEF – left ventricular ejection fraction, NYHA – New York Heart Association, M – male, RBBB – right bundle branch block, RV – right ventricular. Scar on DE-CMR depicted in % of LV mass.*


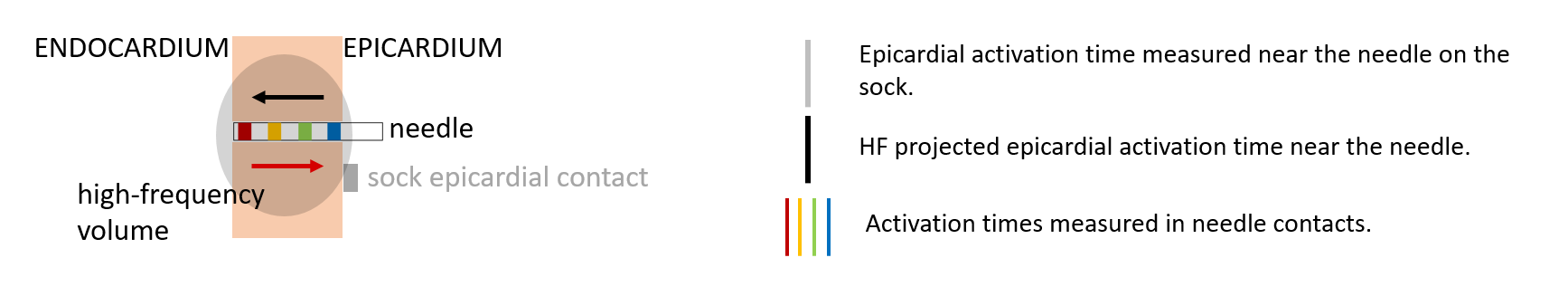


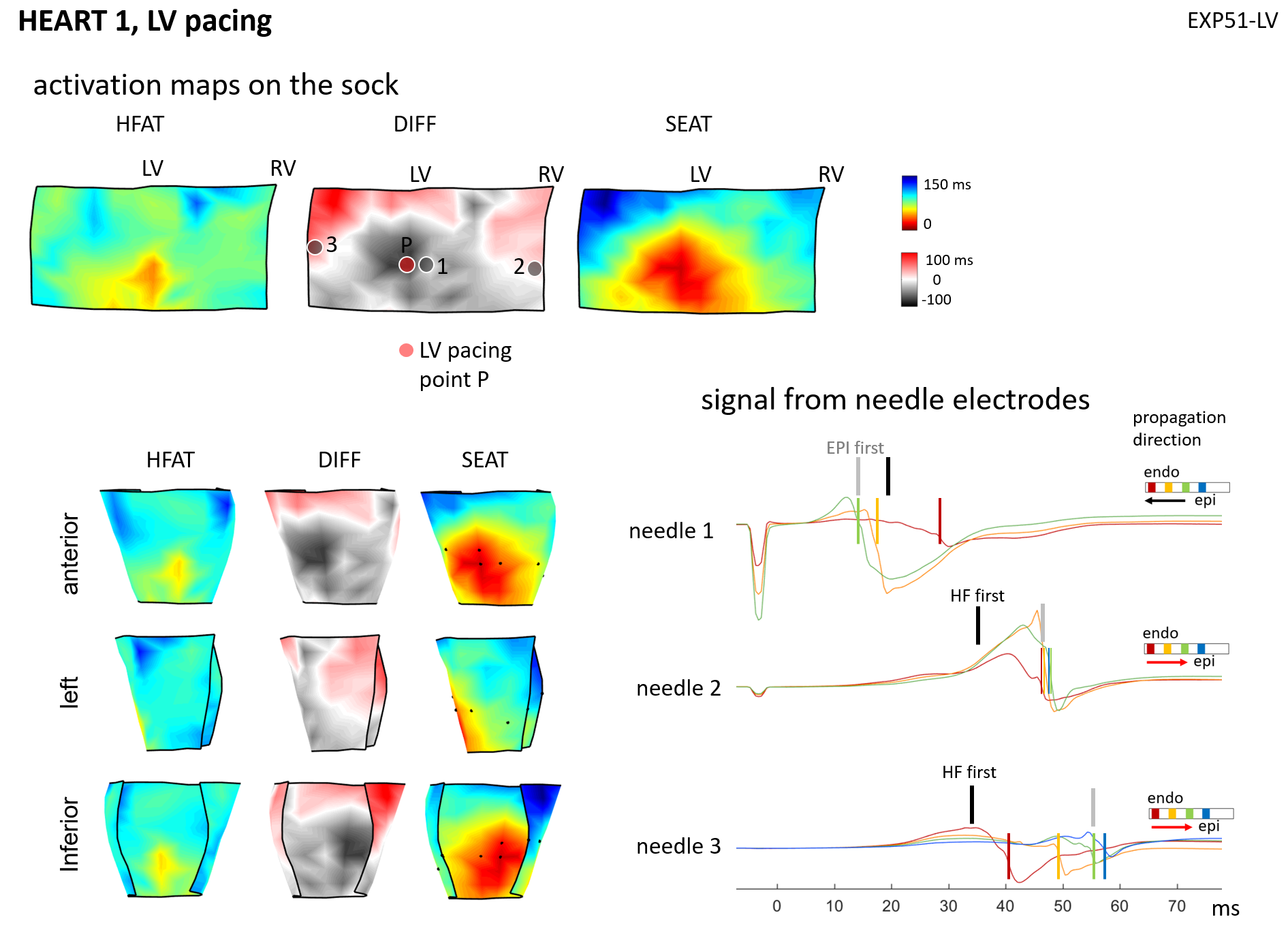


***Figure S1.*** *Ex-vivo data – Heart 1, left ventricular epicardium pacing.*

*Pig heart, perfused in Langendorff mode, 256 torso electrodes, 108 sock electrodes, three plunge electrode needles - each 4 contacts.*

*Left panel: SEAT - activation map on the sock, HFAT - high-frequency activation map, DIFF - differential activation map SEAT-HFAT,*

*Right panel: transmural depolarization wavefront propagation using signals from needle electrodes. Each needle electrode contained 4 contacts, marked by color (Blue= close to epicardium; red = close to endocardium). The vertical color lines in the needles potentials indicate related activation time being the maximum negative derivative in each signal.* *The position of the electrodes is indicated in DIFF map.*

*The gray and black vertical marks define the activation time measured on the sock close to the needle electrode (SEAT) and the activation time calculated by the HFECGI method again close to the needle electrode (HFAT), respectively.* *Red and black arrows point to a predominant endocardium to epicardium and epicardium to the endocardium propagation direction.*


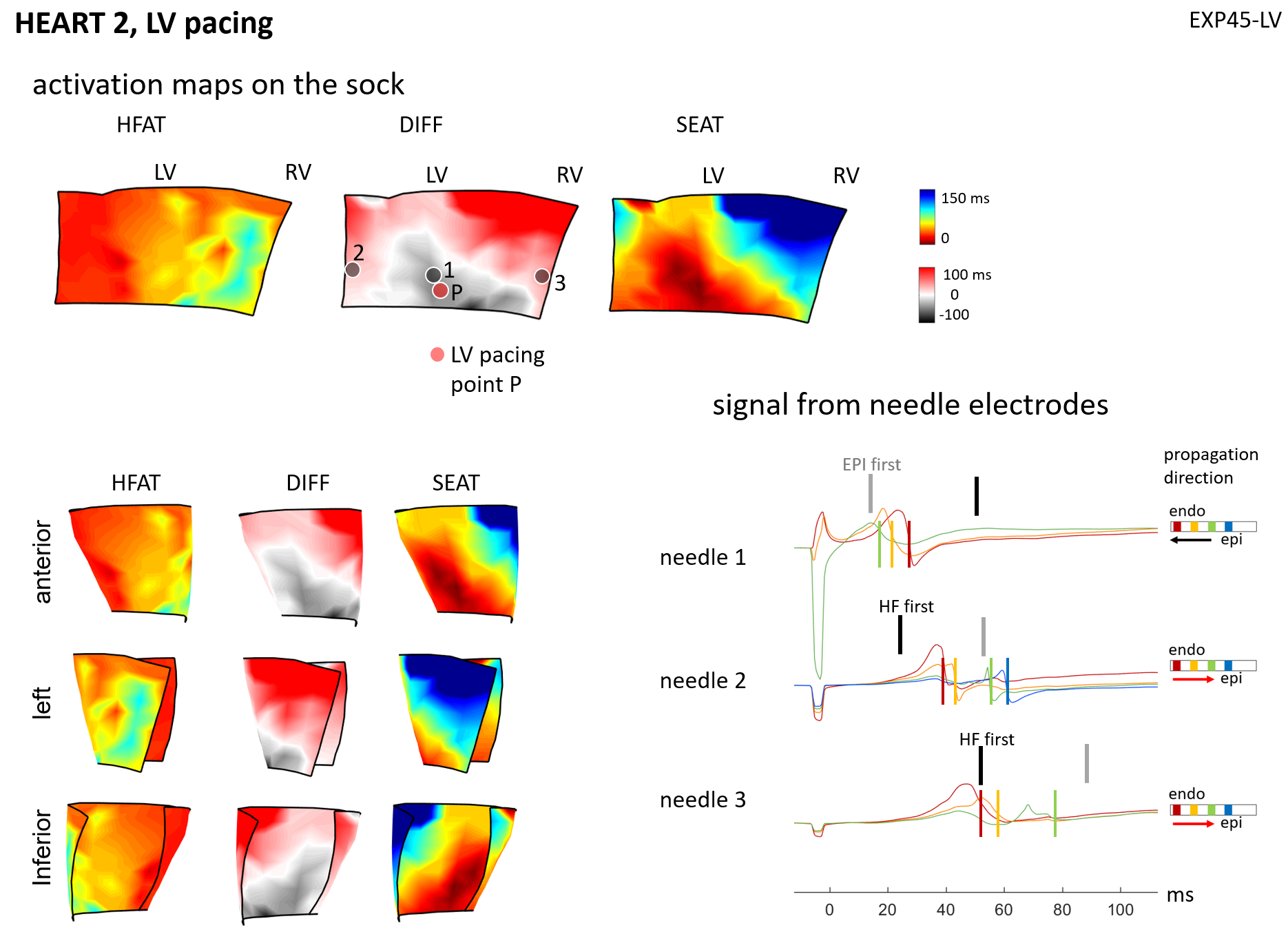


***Figure S2.*** *Ex-vivo data – Heart 2, left ventricular epicardium pacing*


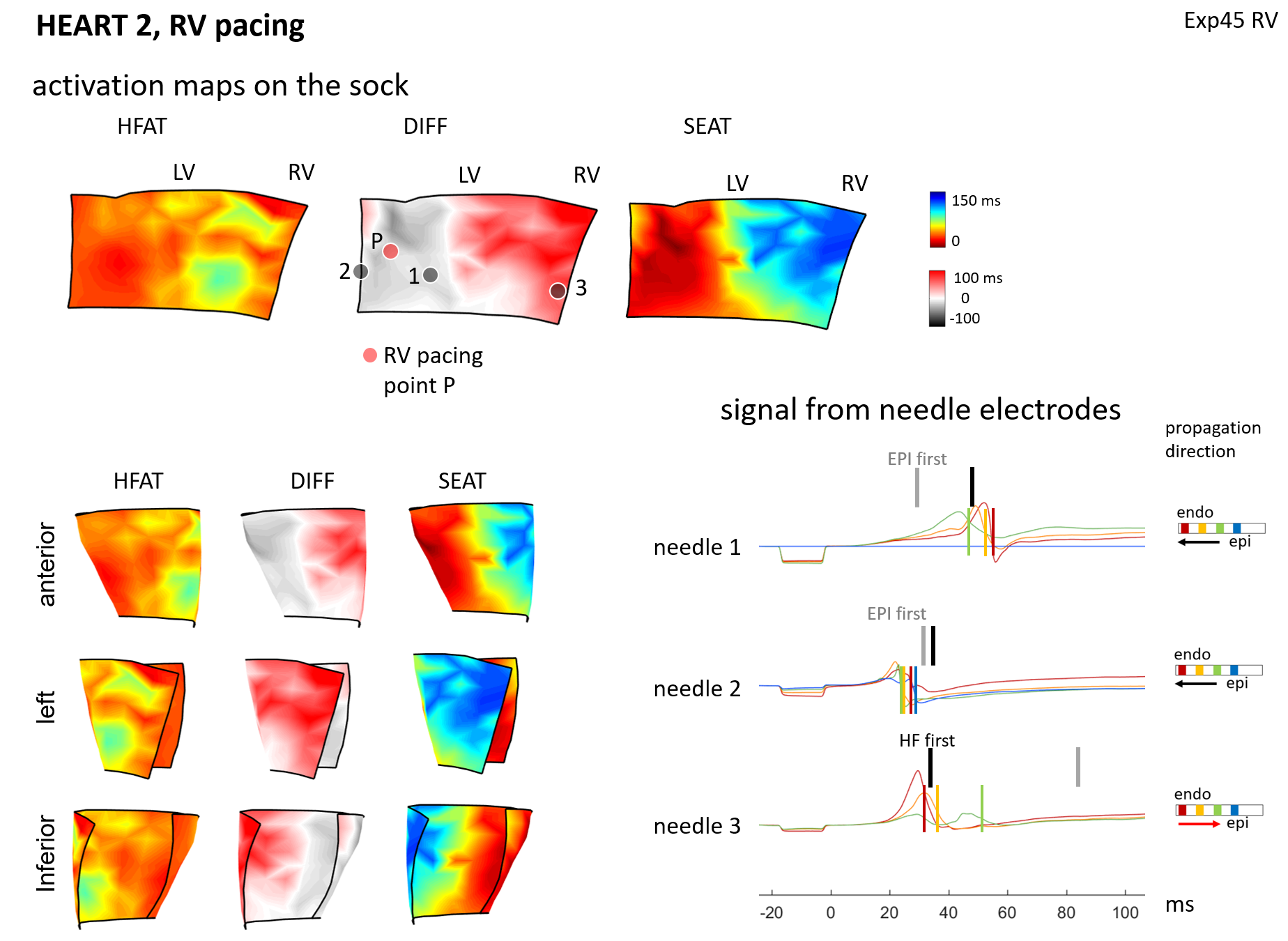


***Figure S3.*** *Ex-vivo data – Heart 2, right ventricular epicardium pacing*


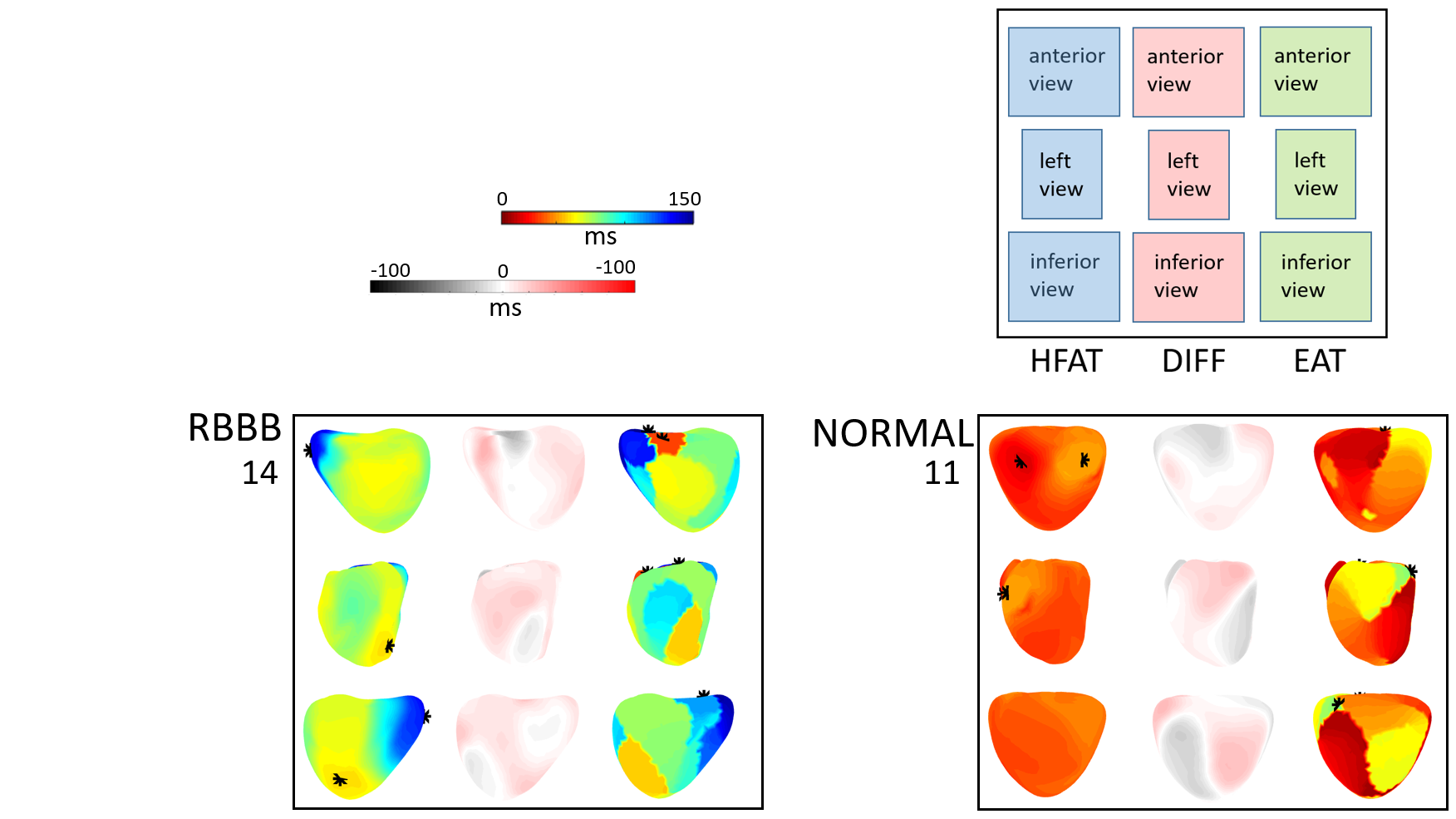


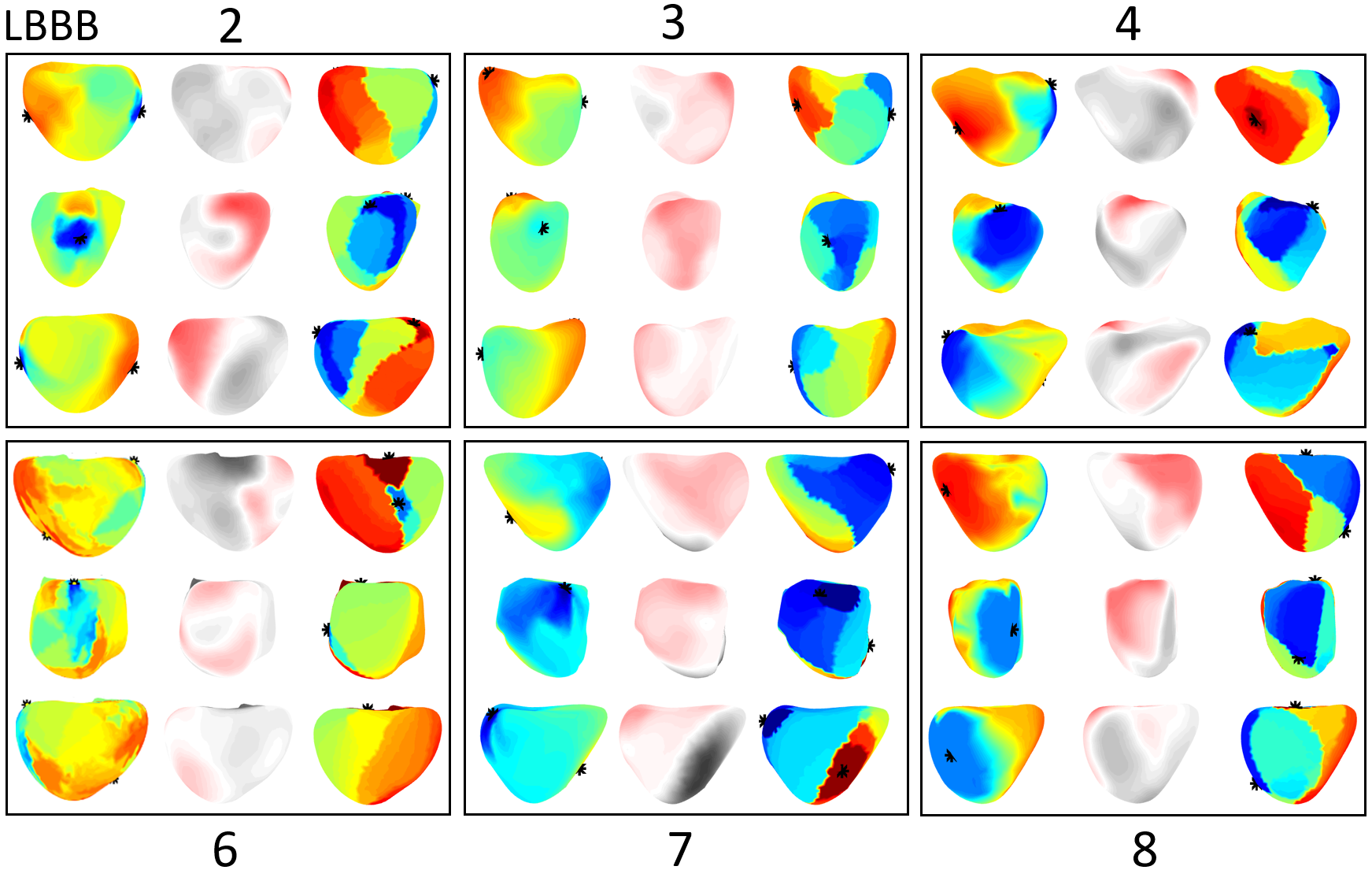


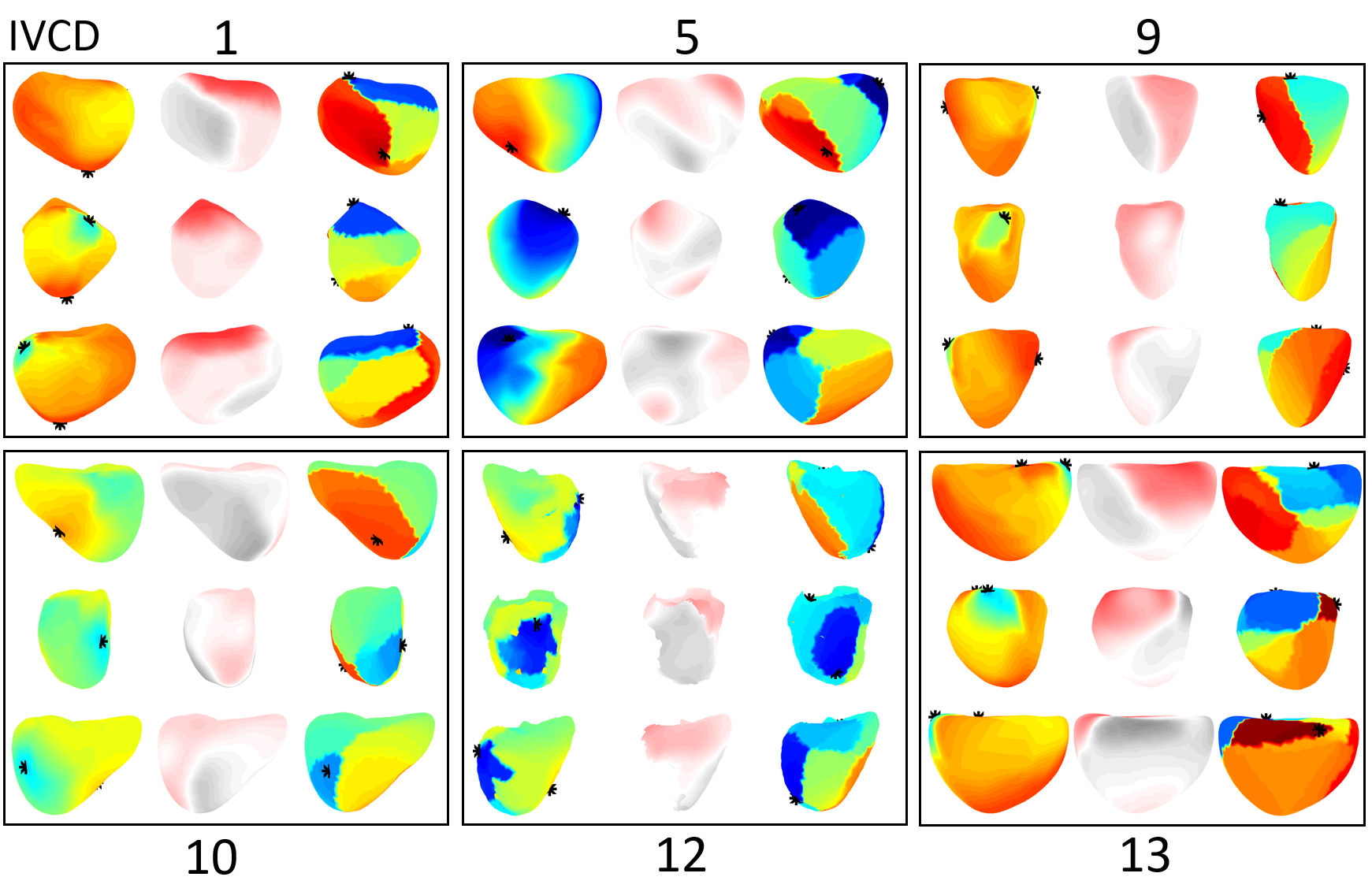


***Figure S4.*** *High-frequency activation times (HFAT), epicardial activation times (EAT) and differences (DIFF), complete results for 14 patients listed in Table 1. Upper panel: Description of image layout for a single patient – each map is shown from three projections (anterior, inferior, and left), Right bundle branch block (RBBB) patient, and patient with normal synchronous heart. Middle panel: Left bundle branch block (LBBB) patients. Bottom panel: intraventricular conduction disturbance (IVCD) patients.*


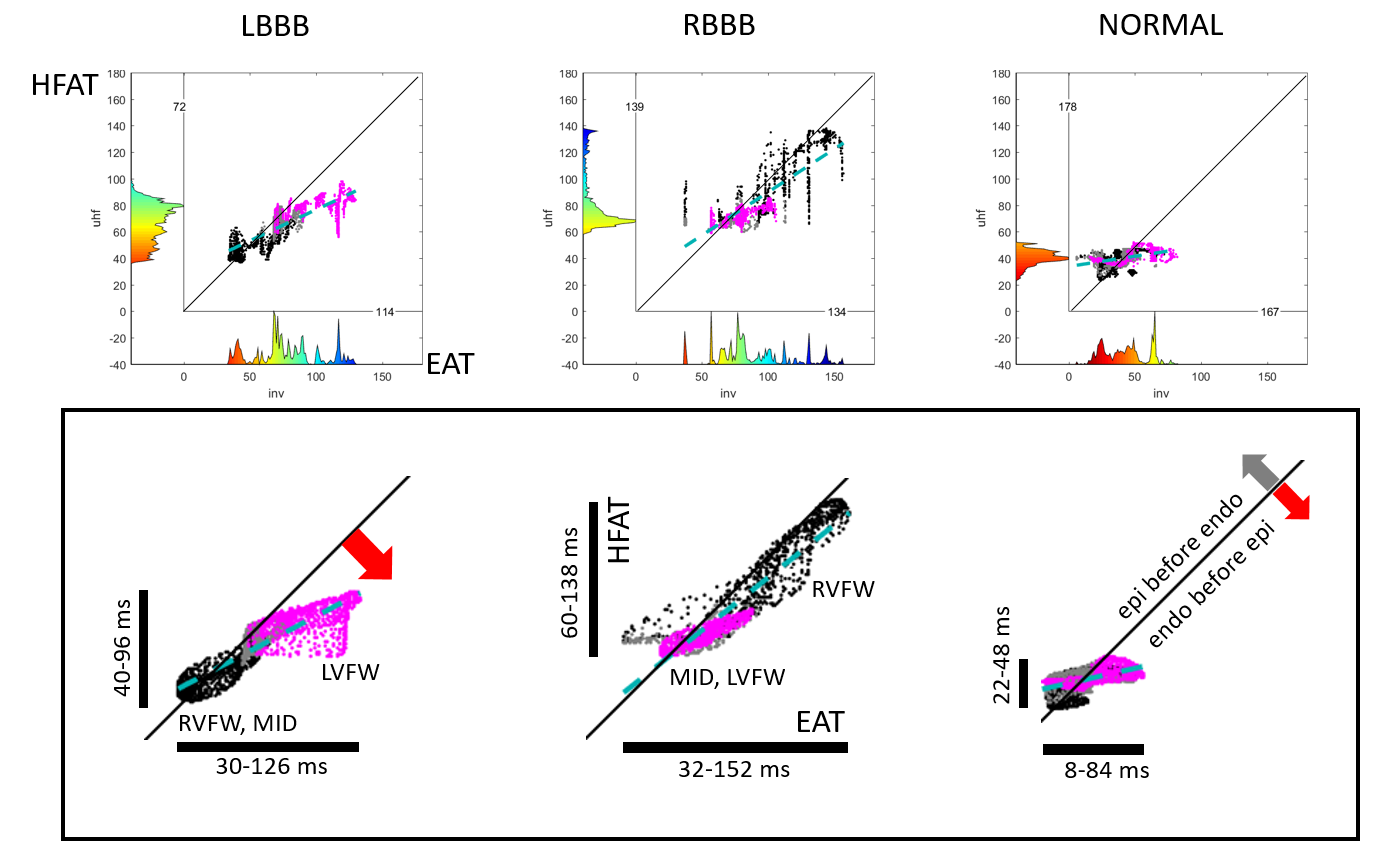


***Figure S5.*** *Scatter plots of EAT vs. HFAT, in unsmoothed (upper) and smoothed form (lower). Smoothing was performed over six surrounding virtual points. The graphs along the X and Y-axis indicate the number of points at a certain EAT and HFAT value.*

*Virtual points identification: Black – right ventricular free wall, violet – left ventricular free wall, grey – mid septal part.*


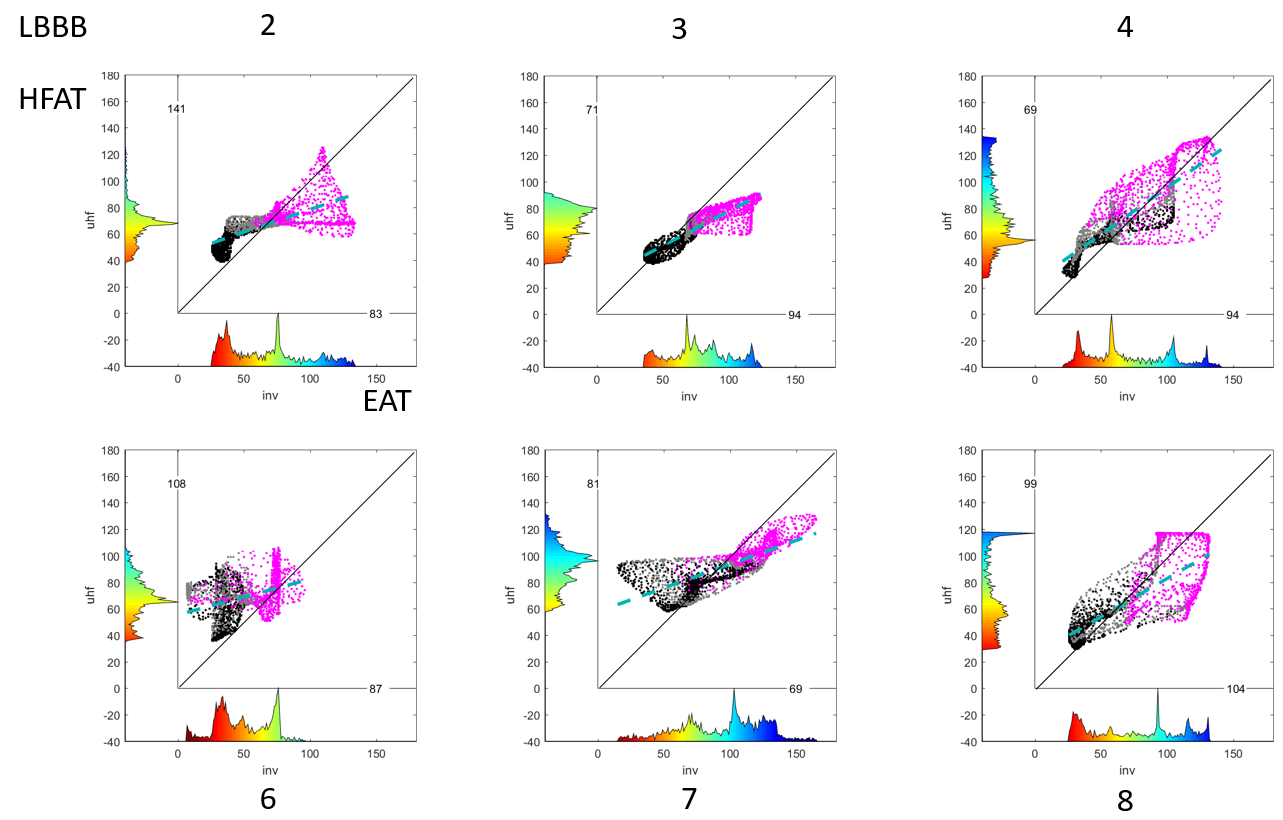


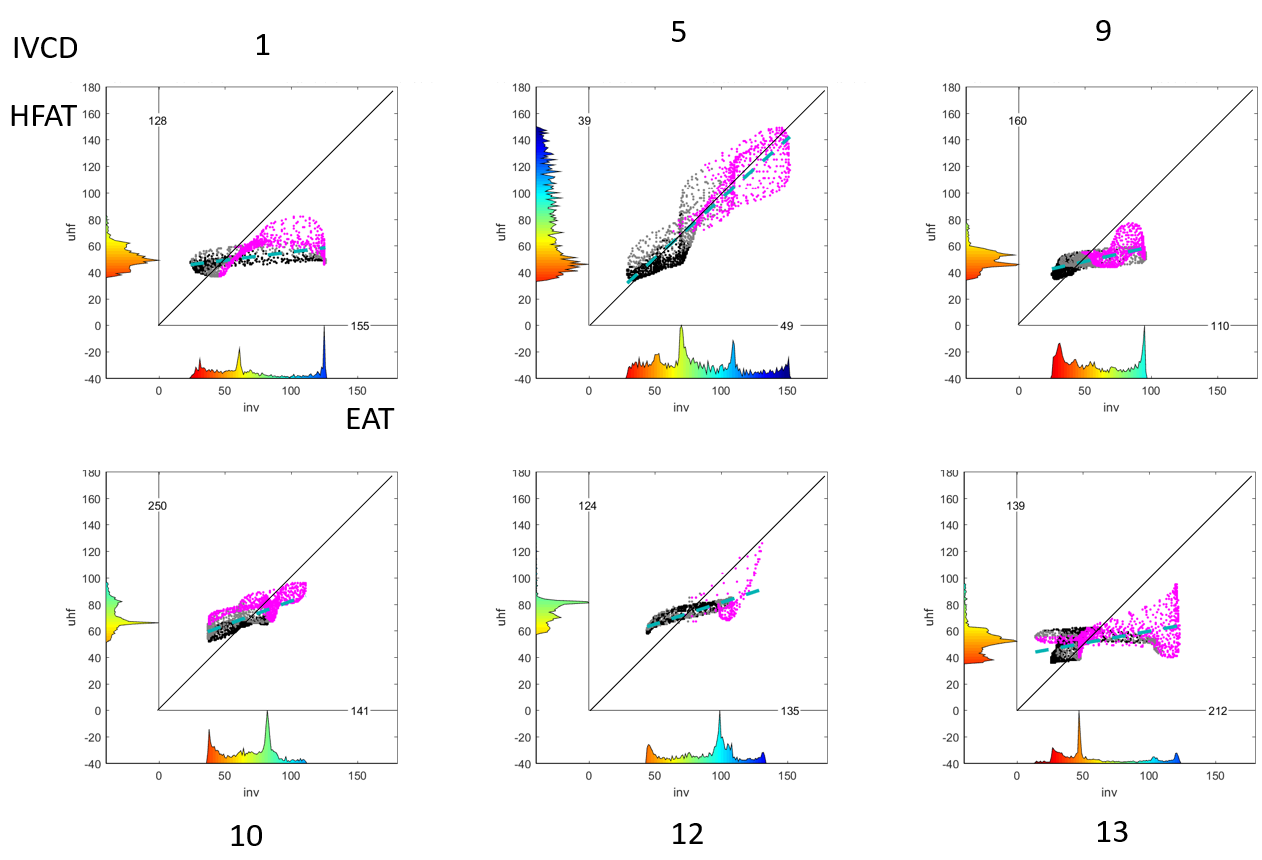


***Figure S6****. EAT and HFAT scatter plots of all LBBB and IVCD patients.*

**Methods**

**HFECG projection on the epicardium**

HFECGI uses a direct projection of the HFQRS body surface on the epicardium. It differs from ECGI, which uses inverse reconstruction. The following Figure shows the direct projection method. An advantage of direct projection is the ability to reduce the number of body surface electrodes.


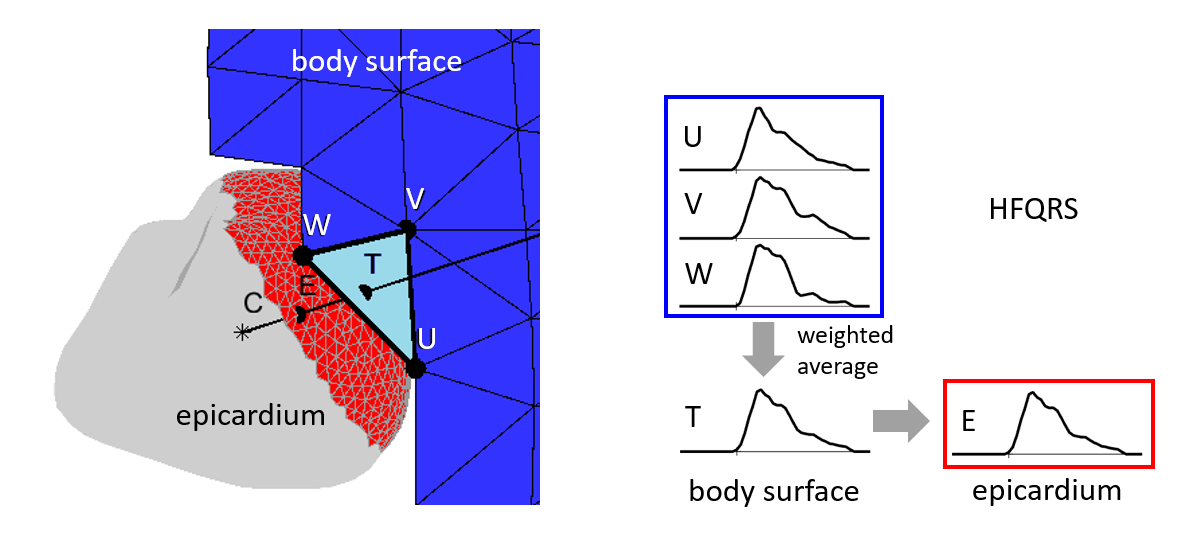


***Figure S7. Direct projection of body surface HFQRS on to heart epicardium.***

***Left panel.*** *C - heart geometry center, E - the virtual point on the epicardium, T - the body surface point where crossing the line originated in heart geometry center C and passing the virtual point on epicardium E.*

***Right panel.*** *The T point does not directly intersect the surface electrode. Therefore, the weighted average of three HFQRSs from three T point nearest electrodes (U, V, W) is used. The electrode distance from point T determines the weight.*

**Frequency range and HFQRS**

The frequency range of an ECG is usually used in the bands below 50/60 Hz. Frequencies above this limit are used, for example, for the detection of late potentials (50-300 Hz), QRS fragmentation (up to 100 Hz), or for the diagnosis of ischemia in the high-frequency band (150-250 Hz). Frequencies above this limit have not been used so far. Nevertheless, information can also be found in higher bands - see Figure S8. The distribution of signal amplitudes above 100 Hz involves variability in different frequency bands, Figure S8, right panel. It is not yet known whether this variability provides useful information. It will be the subject of further research.


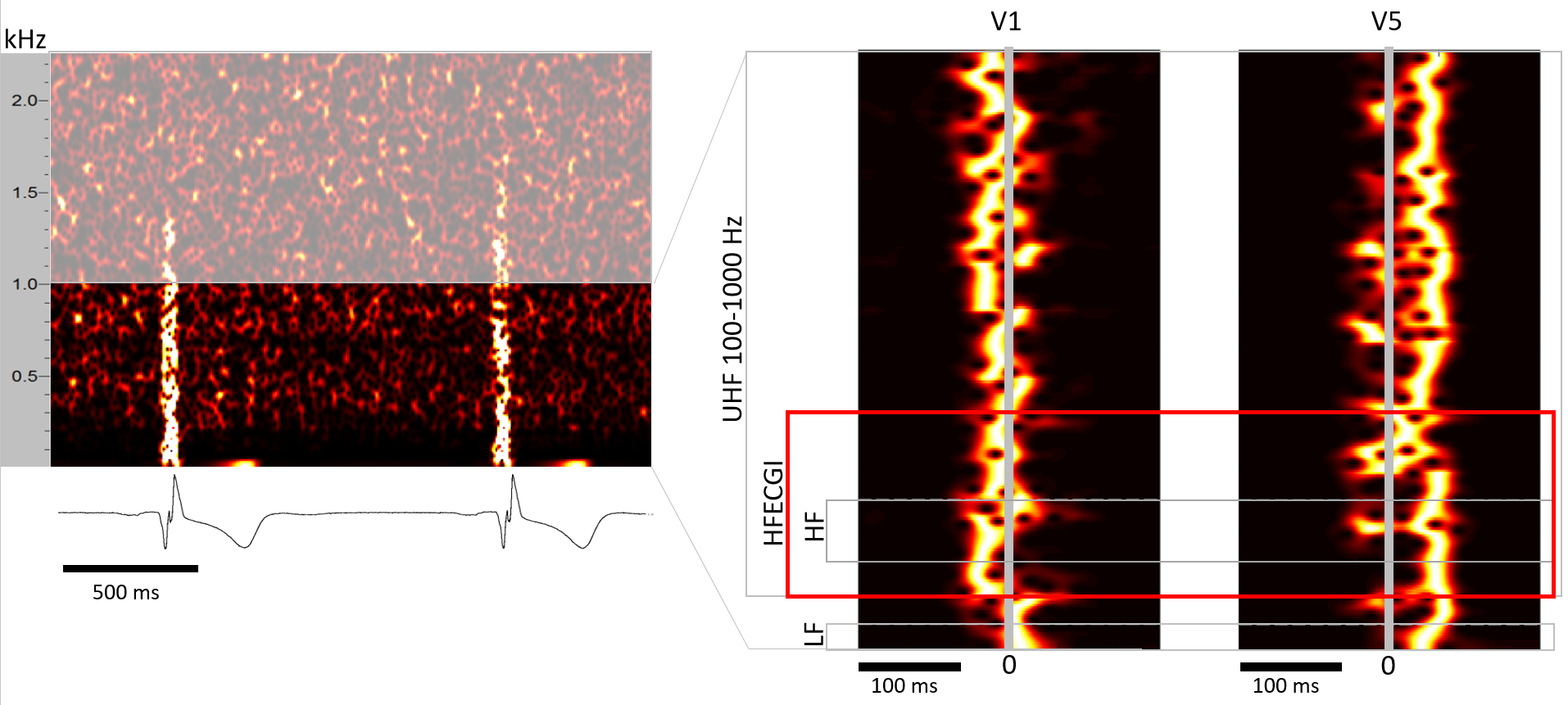


***Figure S8. The frequency ranges of the QRS complex.***

***Left panel:*** *Time-frequency map of two consecutive beats. The horizontal axis shows time; the vertical axis shows frequency (0-2300 Hz). The color shows the normalized amplitude of the HF components (black 0, white 1). In this case, the frequency content of QRS complexes up to 1.5 kHz can be identified. The BioSDA09 (M&I Prague) acquisition unit, 25kHz sampling frequency, 24 bits, 10 nV/bit, 0.1-2 kHz low pass filter, down sampled to 5kHz.*

***Right panel:*** *Time-frequency map averaged from 200 QRS complexes. The horizontal axis shows time; the vertical axis shows frequency (0-1000 Hz). The color shows the normalized amplitude of the HF components (black 0, white 1), leads V1 and V5. The following frequency bands are marked: LF 0-40 Hz, HF 150-250 Hz, HFECGI 100-350(400) Hz, and UHF 100-1000 Hz. It can be seen that the difference between HF and LF information starts somewhere in the 80-100 Hz region.*

**Frequency averaging**

Averaging in different frequency bands eliminates the frequency-dependent variability of HFQRS – Figure S8, right panel. For frequency averaging, we compute HFQRSs in floating 100 Hz windows – 100-200, 150-250, 200-300, 250-350, 300-400 Hz.

The amplitude of HFECG decreases with increasing frequency. In order to perform frequency averaging, the amplitudes in different frequency bands must be normalized. Normalization makes it possible to average low and high frequencies, although higher frequencies have a significantly lower amplitude. Thus, normalization increases the contribution of the low-amplitude signal. So far, we do not know whether different frequency bands provide different information. In the band 100-1000 Hz, we did not notice any regularity associated with various pathologies. Averaging over frequencies, thus eliminates HF amplitude variability and provides stable and clearly drawn HFQRS amplitudes, more details in [13].
